# Application of Nanomaterials in Early Imaging and Advanced Treatment of Atherosclerosis

**DOI:** 10.1021/cbmi.4c00064

**Published:** 2025-01-21

**Authors:** Qianru Zhou, Yujie Wang, Guangxiang Si, Xingbiao Chen, Dan Mu, Bing Zhang

**Affiliations:** † Department of Radiology, Nanjing Drum Tower Hospital Clinical College of Traditional Chinese and Western Medicine, Nanjing University of Chinese Medicine, Nanjing 210008, China; ‡ Department of Radiology, Nanjing Drum Tower Hospital Clinical College of Jiangsu University, Nanjing 210008, China; § Jiangsu Key Laboratory for Biomaterials and Devices, School of Biological Science and Medical Engineering, Southeast University, Nanjing 210000, China; ∥ Clinical Science, Philips Healthcare, Shanghai 200233, China; ⊥ Department of Radiology, Nanjing Drum Tower Hospital, Affiliated Hospital of Medical School, Nanjing University, No. 321 Zhongshan Road, Nanjing 210008, China

**Keywords:** atherosclerosis, molecular mechanism, imaging, treatment, nanomaterials, plaque, cardiovascular disease, molecular

## Abstract

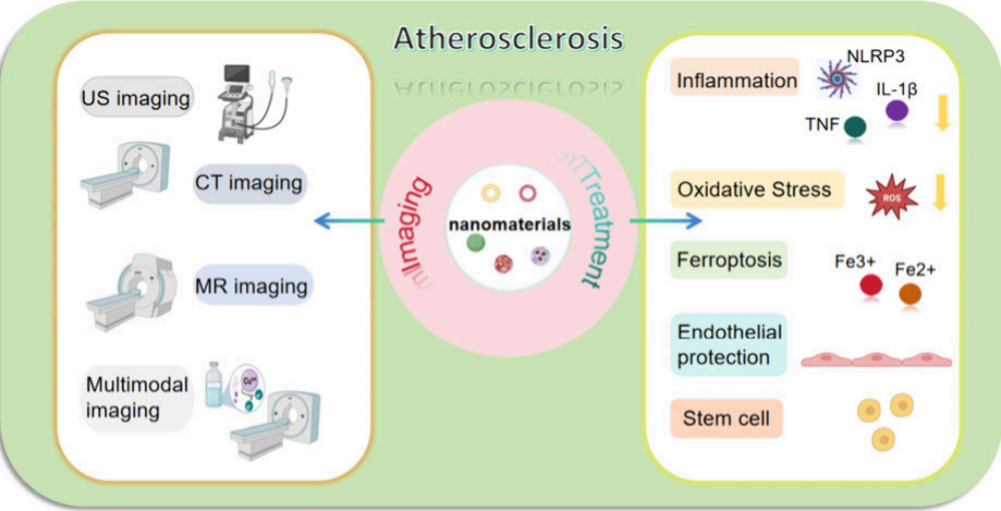

Atherosclerosis (AS) is a serious disease that poses a significant threat to the global population. In this review, we analyze the development of AS from multiple perspectives, aiming to elucidate its molecular mechanisms. We also focus on imaging techniques and therapeutic approaches, highlighting the crucial role of nanomaterials in both imaging and therapy for AS. By leveraging their compatibility and targeting capabilities, nanomaterials can be integrated with traditional medical imaging and therapeutic agents to achieve targeted drug delivery, controlled release, and precise localization and imaging of atherosclerotic plaques.

## 1 Introduction

Cardiovascular disease (CVD) is a major global health issue and the leading cause of death among Chinese residents. In 2019, over 17 million deaths were attributed to CVD.^1,2^ The 2022 China Cardiovascular Health and Disease Report indicated that the prevalence of unhealthy lifestyles and the accelerating population aging have led to a significant population in China with CVD risk factors, with incidence and mortality rates continuing to rise. Projections suggest that the number of CVD patients in China may reach 330 million, exacerbating the burden of CVD. Coronary atherosclerotic heart disease (CHD) is a primary contributing factor, with atherosclerosis (AS) being the main cause of CHD.^3,4^ Annually, AS accounts for half of global mortality. Consequently, global medical organizations persist in researching the pathogenesis and treatment of AS, aiming to enhance prevention and treatment strategies and reduce morbidity and mortality rates worldwide.^5^

The symptoms of AS primarily depend on vascular lesions and the degree of ischemia in the affected organs. Coronary atherosclerosis can lead to angina pectoris, myocardial infarction, arrhythmia, or even sudden death if the diameter of the coronary artery is narrowed by more than 75%.

In response to this serious situation, doctors often use drugs to control the treatment, for very serious blood vessel blockage need to take surgical means of treatment, these conventional treatments are mainly to alleviate the symptoms of atherosclerosis, slow down the development of the rate, so as to prolong the patient’s survival time. However, these methods have some drawbacks, such as drug resistance, side effects, or the risk of trauma and complications.

In recent years, the development of nanomaterials has accelerated as researchers have recognized their significant potential in enhancing disease diagnosis and imaging. This review explores novel diagnostic and therapeutic approaches to effectively treat diseases using nanomaterials by various research teams in recent years, with a focus on atherosclerosis. It not only presents traditional imaging and therapeutic techniques for atherosclerosis but also highlights the critical roles of various nanomaterials in treating the condition. This review examines these nanomaterials from the perspectives of disease pathogenesis and molecular mechanisms of action and discusses their future potential in plaque imaging and therapy, aiming to improve the effectiveness of disease treatment.

## 2 The Molecular Mechanism and Pathogenesis of Atherosclerosis

**Figure 1:**
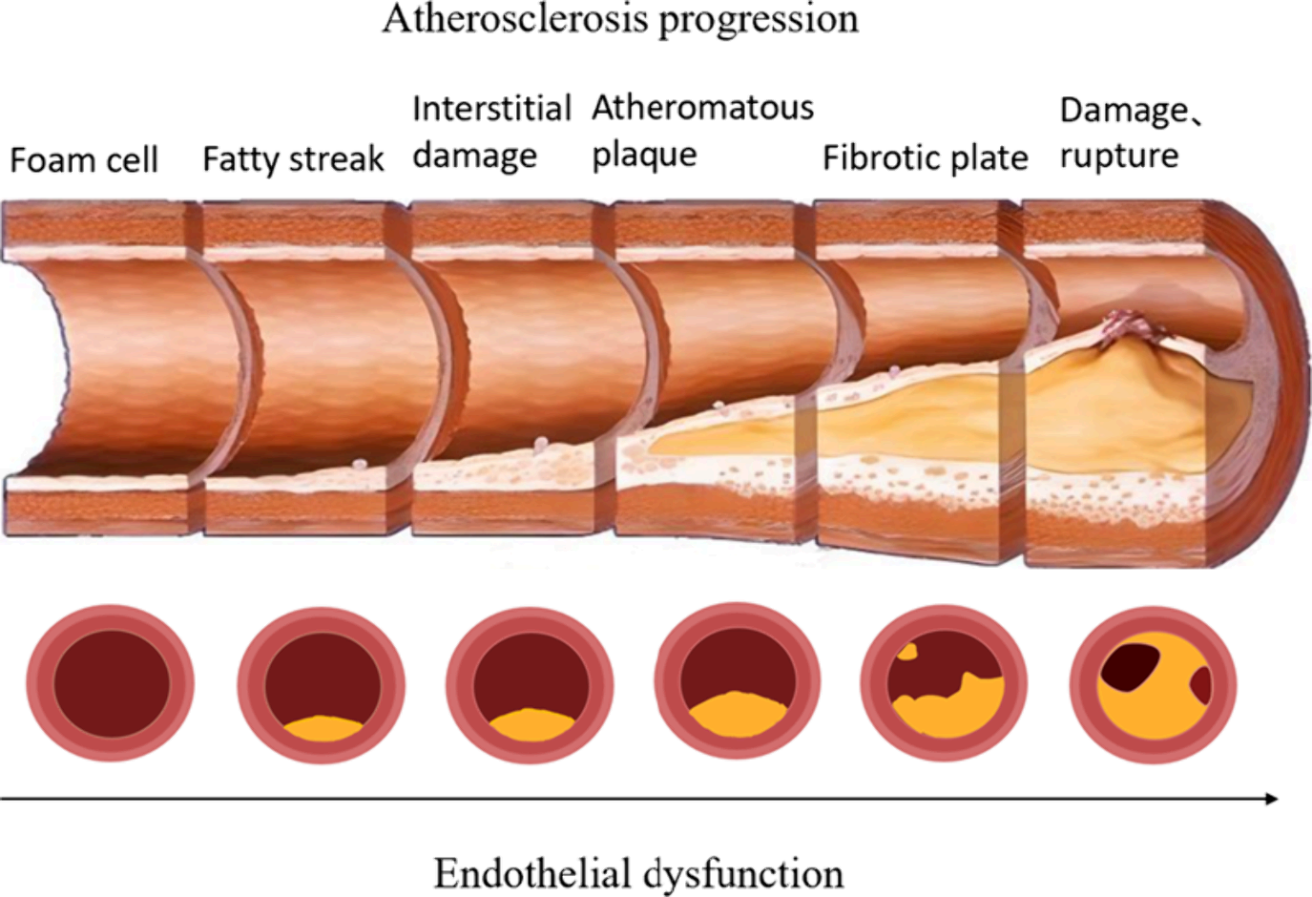
Endothelial Dysfunction in Atherosclerosis. The cross-sectional and longitudinal views of the vessel demonstrate that as plaque forms and expands, the lumen narrows, reducing the space available for blood flow.

Atherosclerosis is the formation of atheromatous lipid-containing necrotic lesions and hardening of the vessel wall in the intima of the arteries due to the deposition of lipids and other blood constituents, proliferation of smooth muscle cells, and increase in collagen fibers (Figure 1). If left unchecked, secondary lesions such as calcification, atheromatous ulcer formation, thrombosis and intraplaque hemorrhage can occur as the lesions continue to worsen. From the perspective of disease development, Multiple factors contribute to the development of atherosclerosis (AS).

Lipid infiltration leading to the development of AS is one of the hypotheses for the pathogenesis of AS. Other hypotheses include thrombosis and platelet aggregation, injury response, and monoclonalism. These different factors influence the formation and development of AS in different ways. In the lipid infiltration theory, lipids enter the vessel wall through various pathways and are deposited beneath the endothelial cells of the vessels, and the deposited lipids gradually increase to form lipid spots, which then develop into lipid streaks.^6^ As lipid deposition intensifies, smooth muscle cells and macrophages, etc. within the vessel wall begin to proliferate and secrete large amounts of collagen fibers and other matrix components, forming fibrous tissue proliferation, with the fibrous tissue wrapping around the lipids to form the underlying structure of the plaque.^7,8^ At the same time, calcium salts and other substances are gradually deposited within the plaque and on the vessel wall. Calcium deposition further exacerbates the hardening of the vessel wall and the narrowing of the lumen, and the extent and location of calcium deposition has an important impact on the stability of the plaque and the risk of rupture. As lipid deposition, fibrous tissue proliferation, and calcium deposition continue to progress, the structure of the vessel wall changes. The gradual calcification of the arterial intima-media causes the vessel wall to lose its original elasticity and toughness, leading not only to thickening and hardening of the vessel wall, but also to narrowing or even blocking of the lumen, which affects the normal flow of blood. Due to the combined effects of lipid deposition, fibrous tissue proliferation, calcium deposition and structural changes in the vessel wall, the lumen of the arteries becomes narrowed or even occluded, which may lead to insufficient blood supply to vital organs such as the heart and the brain, and lead to angina pectoris, myocardial infarction, cerebral infarction and other serious complications. In summary, atherosclerosis begins with the excessive accumulation of lipids in the endothelium, which leads to plaque formation, blockage and narrowing of blood vessels, thus causing malignant cardiovascular events.^9^

Atherosclerotic plaques are formed by deposits of cholesterol (Ch), lipoproteins, and other cells and molecules. Over time, the plaque grows in size, leading to narrowing and blockage of the arterial lumen. This interferes with the normal delivery of blood and nutrients, leading to a variety of symptoms and diseases associated with cardiovascular disease (CVD). The lipid testing can effectively monitor the concentration of triglycerides(TG), Ch, low density lipoprotein cholesterol (LDL-C), and high-density lipoprotein cholesterol (HDL-C) in the blood. Early symptoms of A are difficult to detect, and LDL is an important factor in promoting the formation of AS. High levels of lipids such as LDL promote the development of AS. Also, LDL shows high levels after plaque formation. Therefore, regardless of whether plaque is already present or not, control of the lipid profile is an important means of preventing serious cardiovascular events.

The molecular mechanism of disease occurrence is briefly described as follows.

### 2.1 Vascular Endothelial Cell Dysfunction

Atherosclerosis can be triggered by factors such as hypertension, hyperlipidemia, diabetes, tobacco smoke exposure, or other poor lifestyle habits.^10^ Hypertension causes damage to the endothelial cells of the arteries by enhancing the impact of blood on the arterial walls, and the damaged endothelium triggers an inflammatory response that further aggravates vascular damage. Hyperlipidemia patients have a slower blood circulation due to elevated concentrations of fat and cholesterol, resulting in a decrease in the oxygen-carrying capacity of the blood and accelerated damage to the mucous membranes lining the walls of the blood vessels. The immune system of diabetics is in a state of hyperactivity and is prone to inflammatory reactions. The inflammatory response promotes vascular endothelial cell damage. Harmful substances such as nicotine and CO_2_ produced during smoking can damage the endothelial cells of blood vessels, causing the integrity of the blood vessel wall to be undermined. These factors damage or even rupture the endothelial cells, increasing the permeability of the vascular endothelium to blood lipids, particularly LDL, which accumulates more readily under these conditions.

Protein kinase C (PKC) expression levels are upregulated in conditions such as inflammation and hypertension. It usually leads to the activation of RhoA/Rho kinase-dependent signaling and the phosphorylation of occluding, which weakens the endothelial barrier.^11^ In response to PKC stimulation, the protein phosphatase 1 regulatory subunit 14A (PPP1R14A), an inhibitor of smooth muscle actin phosphatase, is activated. This activation results in cytoskeletal rearrangement, disruption of cell–cell contacts, and increased permeability.^12^ Consequently, lipid molecules can more easily enter the subendothelial space.

### 2.2 Lipid Oxidation and Accumulation

Cholesterol is often present in the blood as lipoproteins, and plasma LDL is the main carrier of endogenous cholesterol transport, which is degraded and transformed by binding to the low-density lipoprotein receptor (LDL-R) on its cell membrane.^13^ Cardiovascular risk factors lead to enhanced production of ROS generated by NADPH oxidase, xanthine oxidase, mitochondrial electron transport chain (ETC) and dysfunctional endothelial nitric oxide synthase (eNOS). Oxidative stress occurs when there is an imbalance between oxidative and antioxidant effects in the body, favoring oxidation.^14−16^ In the presence of increased oxidative stress in the arterial endothelium, LDL and other substances are oxidized to oxidized low-density lipoprotein (ox-LDL) by myeloperoxidase (MPO), hydrogen peroxide (H_2_O_2_), and other strong oxidative agents. Ox-LDL can activate endothelial cells and trigger both inflammatory and immune responses, promoting the development of atherosclerosis.

### 2.3 Environment in Which Inflammation and Immune Reactions Occur

Excessive accumulation of ox-LDL leads to the activation of vascular endothelial cell adhesion molecules (VCAMs),^17^ recruiting and aggregating inflammatory cells, such as monocytes and T cells, at the lesion site. CD4+ T cells are activated and differentiate into effector T cells, including helper type 1 (Th1) cells, which produce interferon γ (IFN-γ) and other inflammatory factors.^18^ Monocytes in the vascular lumen adhere to the endothelium, penetrate through endothelial gaps, and are activated into macrophages by ox-LDL and other agonists, releasing additional inflammatory factors.

### 2.4 Formation of Foam Cells

**Figure 2:**
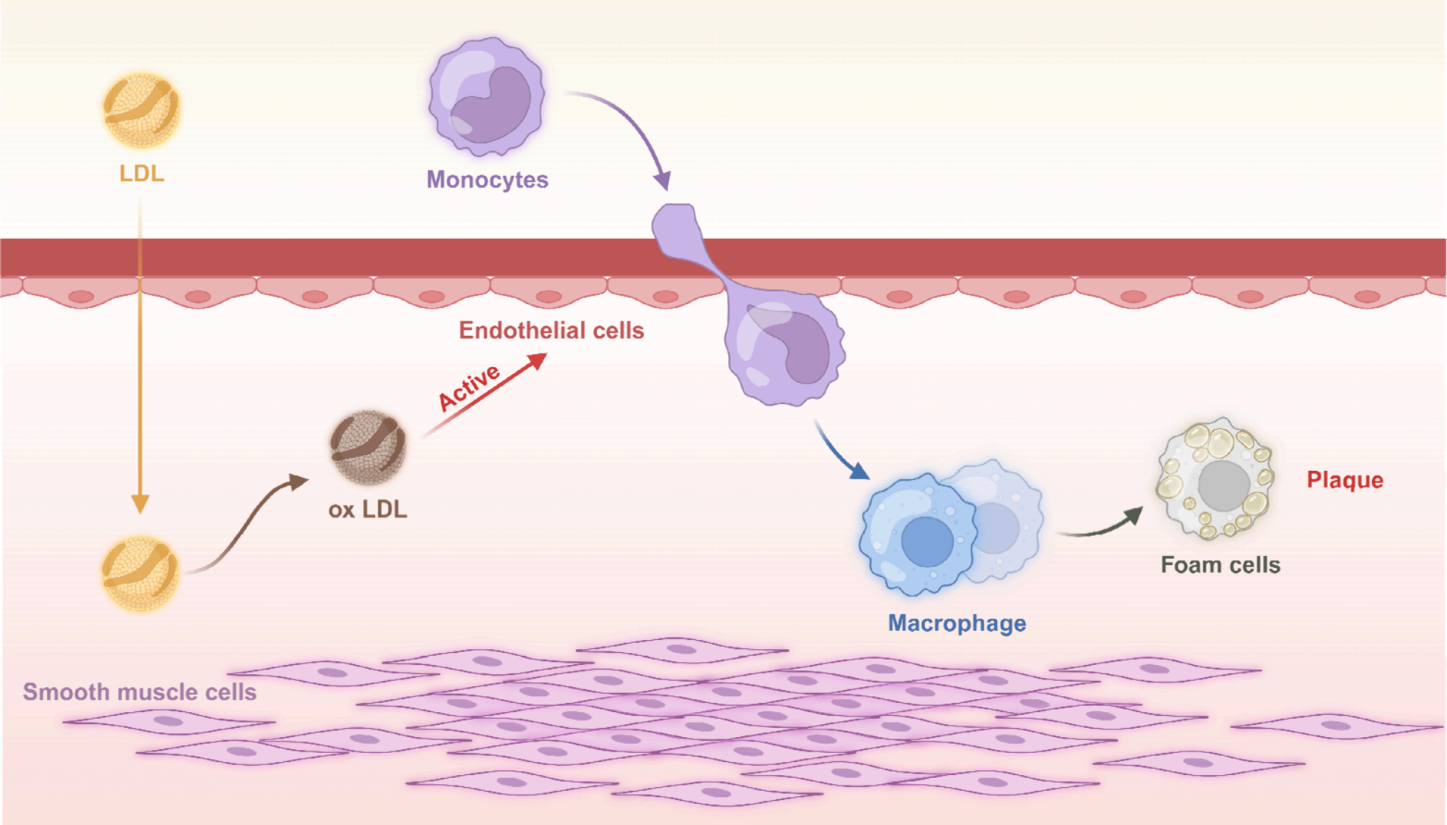
Atherosclerotic Plaque Formation. Low-density lipoprotein (LDL) enters the subendothelial space and is oxidized to oxidized LDL. The accumulation of oxidized LDL stimulates endothelial cells to produce inflammatory factors. Monocytes are recruited and accumulate in the subendothelium, where they are activated by inflammatory cells to become macrophages. These macrophages phagocytose surrounding lipid molecules, transforming into foam cells. These foam cells accumulate in the vascular wall to form fibrous plaques. Smooth muscle cells then migrate to form a fibrous cap, enhancing the stability of the plaque.

Macrophages ingest large amounts of ox-LDL and other liposomes in blood vessels, transforming into foam cells (Figure 2). Foam cell formation is a key factor in the development of atherosclerosis.^19^

Studies have identified several scavenger receptors on the surface of macrophages, such as SR-A1, CD36, and lectin-like oxidized low-density lipoprotein receptor-1 (LOX-1), which are activated or upregulated by various factors and exhibit strong affinity for ox-LDL. These receptors facilitate the uptake of environmental ox-LDL into macrophages, leading to its accumulation and the formation of foam cells. Pro-inflammatory cytokines stimulate nuclear factor-κB (NF-κB),^20,21^ which enhances the expression of SR-A1 and further mediates the phagocytosis of ox-LDL by macrophages, exacerbating foam cell formation.

CD36, a B-type scavenger receptor, also has a strong affinity for ox-LDL.^22^ The expression of CD36 in macrophages is influenced by several factors.^23^ In monocytes, chondroitin activates CD36 expression through the induction of serine production.^24^ Higher levels of CD36 bind ox-LDL more efficiently, promoting macrophage phagocytosis.

The level of LOX-1 is influenced by external factors, such as elevated ox-LDL levels and pro-inflammatory factors, which stimulate LOX-1 expression. This further mediates macrophage uptake of lipids and intensifies the process of foam cell formation.

**Figure 3:**
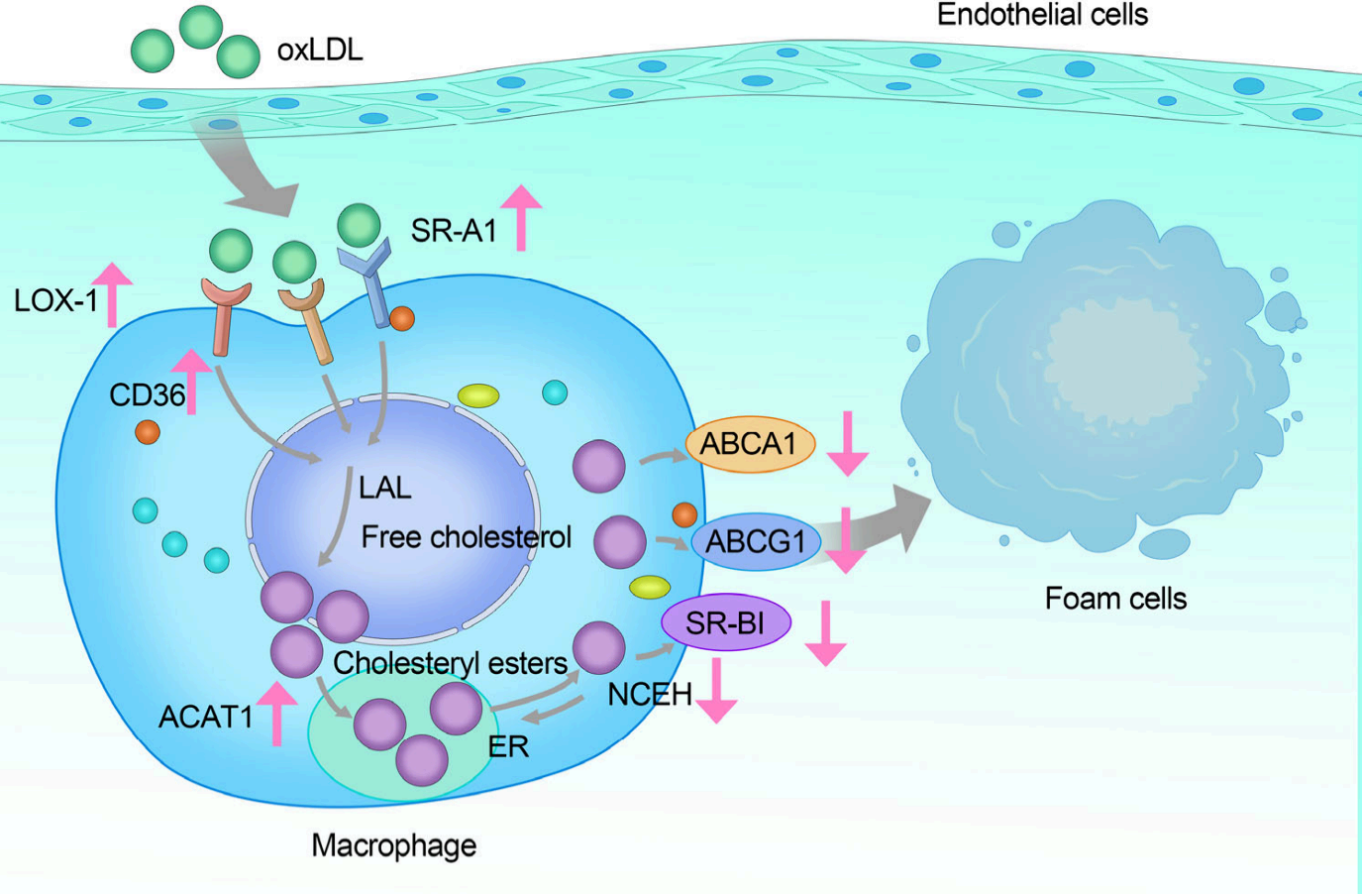
Mechanisms of Lipid Handling in Macrophages. When excess ox-LDL damages the endothelium and enters the subendothelial space, it is phagocytized by macrophages via receptors such as LOX-1, CD36, and SR-A1, which are highly expressed on macrophages. In lysosomes, cholesteryl esters in LDL are broken down into free cholesterol by lysosomal acid lipase (LAL). At this point, cholesteryl ester accumulation predominates due to high expression of acetyl coenzyme A acetyltransferase 1 (ACAT1) and low levels of NCEH. Increased scavenger receptor expression leads to enhanced ox-LDL uptake by macrophages, while the expression of cholesterol transporters such as ABCA1, ABCG1, and SR-BI is relatively low. This imbalance results in the accumulation of cholesteryl esters in macrophages, further promoting their transformation into foam cells.

Under normal conditions, macrophages metabolize intracellular free cholesterol through various transporter carriers, such as ATP-binding cassette transporter A1 (ABCA1), ATP-binding cassette transporter G1 (ABCG1), and scavenger receptor class B type I (SR-BI), or store it in the cytoplasm as ester droplets. When excess lipids enter macrophages, this balance between lipid uptake and clearance is disrupted. Scavenger receptor expression significantly increases, while the expression of transporter carriers is inhibited, blocking cholesterol efflux and leading to increased intracellular deposition. Under abnormal conditions, upregulation of ACAT1 and downregulation of neutral cholesteryl ester hydrolase (NCEH) result in excessive cholesteryl ester deposition in macrophages, further promoting foam cell formation (Figure 3).

Overall, intravascular ox-LDL has a strong affinity for scavenger receptors on the surface of macrophages, thus promoting phagocytosis of ox-LDL by macrophages. Meanwhile, when excess lipid enters macrophages, the expression level of scavenger receptor increases, ACAT1 is upregulated, and NCEH decreases, whereas the expression of transporter carriers is inhibited, and cholesterol efflux is impeded, leading to an increase in intracellular cholesterol deposition, which collectively promotes the formation of foam cells.

### 2.5 Formation of Fibrous Plaques

Over time, foam cells, cell debris, extracellular matrix components, and smooth muscle cells (SMCs) accumulate in the vascular wall, forming fibrous plaques. The migration and proliferation of SMCs, along with the accumulation of collagen and other matrix proteins, contribute to increased plaque stability.

### 2.6 Plaque Stability and Complications

Plaque stability determines the risk of complications. Plaques can be divided into stable and unstable plaques. Stable plaques have thick fiber caps and small lipid cores and do not rupture easily. Stable plaques, on the other hand, can cause gradual narrowing of blood vessels, restricting blood flow and potentially leading to tissue ischemia.^25^ Unstable plaques have histological features such as more macrophages and foam cells, larger necrotic centers, thinner fibrous caps and plaque erosion and calcified nodules.^26^ Unstable plaques may rupture, leading to thrombosis and potentially causing myocardial infarction or stroke. The thin fibrous cap of vulnerable plaques is highly susceptible to rupture under dynamic changes in blood flow or blood pressure, leading to thrombosis.^27^ Therefore, identifying and treating vulnerable plaques is essential to prevent major cardiovascular events.^28−30^

## 3 Nanomaterials for Atherosclerosis Imaging and Treatment

Nanomaterials are materials that have certain nanoscale characteristics at least on a one-dimensional scale. Depending on their morphology and structure, nanomaterials can be classified into many different types, such as nanoparticles, nanowires and nanotubes, nanofilms, nanoporous materials, and nanocomposites. For the specific classification of nanomaterials see 3.1.2Table 2 for details. Nanomaterials generally range in size from 1 to 100 nm, and semiconductor nanoparticles smaller than 10 nm are also known as quantum dots due to the quantization of electron energy levels.^31,32^ Nanomaterials are capable of penetrating membrane cells and traveling along nerve cell synapses, blood vessels and lymphatic vessels. At the same time, some nanomaterials can selectively accumulate in different cells and certain cellular structures. It is these properties that allow nanomaterials to be used for research applications in the medical field.

Nanomaterials enter cells in two main ways, phagocytosis and cytophagy. Phagocytosis refers to the uptake of conditioned particulate matter and solutes by cells through vesicles that are micrometer in size. Cytophagy refers to the uptake of fluid containing solutes and particles by vesicles, but the size of vesicles in cytophagy is smaller than those produced during phagocytosis. This mechanism of endocytosis can be categorized into macropinocytosis and receptor-mediated endocytosis (RME).^33^ Macropinocytosis permits the uptake of raw materials through large vesicles, varying in size, called macropinosome. Upon internalization of macropinosomes, the pH gradually decreases and endosomal markers begin to appear. Subsequently, acidified macropinosomes can either fuse with late endosomes, bind to lysosomes or recycle the material they transport to the cell membrane. Receptor-mediated endocytosis is one of the most common routes by which nanoparticles enter the cell interior. Receptor-mediated endocytosis begins when a ligand attached to a nanoparticle binds to a specific receptor on the cell membrane, and the binding triggers a conformational change leading to plasma membrane invagination and the generation of early endosomes. There are different types of RME, one being clathrin-mediated endocytosis (CME), and the other being caveolin-mediated Endocytosis (CVME). There are also pathways that do not require either of these two protein-mediated pathways, but these pathways have less impact on nanoparticle uptake.

Nanomaterials applied to AS generally need to have good biocompatibility and targeting properties, and the surface of nanomaterials can be biologically or physically or chemically modified to improve the targeting ability, which is expected to be utilized for precise targeted imaging and treatment of AS lesion sites.^34,35^ For example, in order to improve the targeting ability of nanomaterials for lesion sites, ligands with strong affinity for the lesion area can be modified on the surface of nanomaterials. Since AS lesion sites have a stronger inflammatory response, increased ROS production, and decreased pH, pH and ROS responsive nanomaterials can also be designed to enhance the targeting effect.

In addition, modification of biomimetic cell membranes on the surface of nanomaterials can significantly enhance their biocompatibility, such as nanoparticles with surface-encapsulated platelet membranes. Since the endothelial cells on the vulnerable surface are activated and damaged, collagen is exposed, and macrophages inside the plaque are overaggregated and easily adhere to platelet membrane proteins, nanoparticles wrapped around platelet membranes can be rapidly targeted and aggregated in the lesion area. As a result, such nanoparticles exert a targeting effect while maintaining excellent biocompatibility and safety.

Liposomes (LNPs) are formed from phospholipids to form closed vesicles with a bilayer structure, which can efficiently load the drug and protect it from metabolic processes. After targeted modification, liposomes can be highly enriched at the site of the lesion and, due to their slow-release characteristics, can improve retention while reducing cellular toxicity and improving safety.^36,37^ Studies on LNPs in cardiovascular diseases have shown that they possess good biocompatibility and biodegradability,^38,39^ and LNPs stabilize compounds for therapeutic use and overcome barriers to cell and tissue uptake.^40−42^

Studies have shown that LNPs can interact with apolipoprotein E (ApoE) APOE and HDL proteins in vivo with strong hepatic targeting. It can reach hepatocytes by passive adsorption of ApoE, leading to LDL-R mediated endocytosis.^43−46^ Therefore, they are often used in liver disease treatment and vaccines. Therefore, the targeting of extrahepatic organs and tissues is of particular importance. Surface modification of liposomes can enhance the targeting of loaded drugs.

HDL consists of apolipoproteins, phospholipids, cholesterol and a small number of fatty acids. HDL particles are small and can move freely in and out of the arterial wall, and can ingest harmful substances such as LDL, cholesterol, and triglycerides that are immersed in the bottom layer of the intima of the vessel wall, and transport them to the liver for catabolism and excretion. Previous studies have demonstrated that HDL can be actively transported into lymphatic vessels via SR-BI expressed on lymphatic endothelial cells.^47,48^ Researchers therefore developed a cancer nanovaccine based on HDL-mimicking LNP, R837/LNP-M-L, capable of efficiently targeting the lymphatic, which packages tumor-specific membrane antigens and encapsulates them with the hydrophobic immunoadjuvant imiquimod (R837) adjuvant for personalized cancer immunotherapy. The optimized nanovaccine promotes codelivery of antigen and adjuvant to lymph nodes and maintains antigen presentation to dendritic cells, resulting in long-term immune surveillance as cytotoxic T-lymphocyte (CTL) frequency rises in lymphoid organs and tumor tissues, which inhibits tumor formation and growth and enhances therapeutic efficacy of checkpoint inhibitors.^49^

By the same token then, it is also possible to design nanomaterials for atherosclerotic diseases based on HDL targets expressed on the surface of macrophages. For example, Diditchenko et al. successfully prepared a novel recombinant high-density lipoprotein (rHDL), CSL112, using phosphatidylcholine (PC) reconstituted apolipoprotein (Apo A-I), and demonstrated that it effectively promotes cholesterol efflux and ameliorates atherosclerotic conditions.^50^ A team of researchers used two components, a single peptide and saturated dimyristoylphosphatidylcholine (DMPC), to construct a peptide-based recombinant high-density lipoprotein (pHDL) by microfluidic means, a nanocarrier with atherosclerosis-targeting properties.^51^ pHDL not only has its own therapeutic effect on atherosclerosis, but also has a unique hydrophilic–hydrophobic structure and a large lipid core, which can be used as a carrier to provide storage space for lipid-soluble drugs, with highly efficient drug delivery. pHDL also has the advantages of small particle size, nonimmunogenic, completely degraded in vivo, and is not recognized by the reticuloendothelial system and is not rapidly cleared, which makes pHDL as a drug carriers show unique advantages in improving plaque-targeted distribution of drugs.

Nandwana et al. successfully mimicked high-density lipoprotein-like magnetic nanostructures (HDL-MNS) resembling natural HDL particles by modifying phospholipids to the surface of the magnetic nanostructures and incorporating apolipoprotein A1 (Apo A1), which can carry HDL.^52^ In contrast to the T2MRI contrast agent Ferumoxytol, HDL-MNS provides 5 times the contrast in 7T MRI. In addition, HDL-MNS functioned in the same way as natural HDL, promoting cholesterol efflux and achieving the efficiency of natural HDL excretion.

LDL nanoparticles are similar to HDL nanoparticles and are highly biocompatible. Similarly, LDL recognizes and binds to LDLR.^53,54^ on the surface of macrophages, which gives LDL nanoparticles natural plaque targeting properties. HDL and LDL nanoparticles are uniquely suited to target plaques, mainly due to the affinity between the relevant gene molecules.

From these, it can be seen that, When combined with gene therapy, nanomaterials can effectively target specific biomolecules in AS plaques, thereby achieving therapeutic effects.

### 3.1 Application of Nanomaterials in the Imaging of Atherosclerosis

#### 3.1.1 Traditional Imaging Methods

AS often presents with rapid onset and subtle early clinical symptoms, making medical imaging essential for diagnosis and treatment. With advancements in science and technology, imaging methods have continuously improved, resulting in enhanced image resolution and greater diagnostic accuracy for AS. These developments have significantly improved diagnostic efficiency.

As shown in Table 1, contemporary imaging techniques have higher imaging resolution and faster imaging speeds, and digital subtraction angiography (DSA) has always been the gold standard for diagnosis in atherosclerotic disease. At the same time, however, traditional imaging techniques have some exposure of patients to radiation and may not be as effective in imaging small vessels.

**Table 1:** Advantages and Disadvantages of Traditional Imaging Methods

Imaging Methods	Advantages	Disadvantages	ref
Ultrasound	1. No radiation exposure	1. Limited for bones and air-filled structures	(55)
	2. Real-time imaging	2. Sensitivity to gas and bone	
	3. High safety	3. Difficulty in imaging deep structures	
	4. Ease of use and cost-effectiveness	4. Potential for artifacts	
	5. Versatility	5. Operator dependence	
MRI	1. No radiation exposure	1. Long scan time	(56, 57)
	2. High resolution	2. Sensitive to patient movement	
	3. Multiparametric imaging	3. Loud noise	
	4. Multiplanar imaging	4. Insensitive to calcification	
	5. Excellent soft tissue contrast	5. Inferior imaging of lung lesions	
	6. No bone artifact		
	7. Versatile for whole-body imaging		
CT	1. High density resolution	1. Ionizing radiation	(58)
	2. Cross-sectional imaging	2. Limited soft tissue contrast	
	3. Reconstructable images	3. Motion artifacts	
	4. High spatial resolution	4. Metal artifacts	
PET-CT	1. Comprehensive imaging	1. Radiation exposure	(59, 60)
	2. High sensitivity and specificity	2. Radiation exposure	
	3. Staging and treatment planning	3. Special considerations	

The limited spatial resolution of ultrasound testing may not allow for detailed visualization of the internal structure of the atherosclerotic plaque. For example, it may be difficult to accurately detect calcification foci smaller than 0.2 mm within the plaque. In addition, ultrasonography has difficulty in showing the spatial status of the plaque vessels and may not be able to reflect the overall condition of the plaque in a comprehensive and accurate manner. Although three-dimensional ultrasound as well as ultrasonography can overcome the deficiencies of two-dimensional ultrasound to a certain extent, there are still some problems. For example, three-dimensional ultrasonography is easily affected by the examiner’s operating experience and variations in the cut surface; ultrasonography lacks a standard evaluation method for analyzing plaque neovascularization, and existing analysis methods suffer from operator variation and poor reproducibility.

In contrast, other imaging techniques such as MRI may have superior performance in some aspects.^61^

For example, MRI is able to sensitively and effectively detect plaques of different natures, visualize vessel walls and perivascular tissues, and is a safe and noninvasive examination. MRI has the highest soft-tissue contrast resolution of all medical imaging tools, and is able to clearly display soft-tissue structures such as muscle, tendon, fascia, and fat. In atherosclerosis imaging, MRI can accurately show structural changes in the vessel wall and its surrounding tissues, which can help to identify pathological changes such as thickening of the vessel wall and plaque formation.^62^ MRI can also differentiate between structures such as plaques and the surrounding vessel wall, and can even show the fibrous cap and lipid core to assess plaque vulnerability. Through the use of specific contrast agents, MRI can also identify the different components of a plaque, thus assessing the stability of the plaque. However, MRI takes longer and is not suitable for patients with pacemakers or metallic implants, and is prone to artifacts.

CT is the opposite of MRI, with CT imaging being short and efficient.^63^ However, CT examination has a certain degree of radiation, although the radiation dose of modern CT equipment has been greatly reduced, but long-term or frequent acceptance of CT examination may still cause potential radiation damage to patients.

In addition, some of the contrast agents used in the imaging process may pose safety risks to the patient.

In general, traditional imaging methods can achieve good results with the assistance of contrast agents. Common contrast agents include I, Gd, and BaSO_4_, each exhibiting distinct adverse reactions over prolonged use.

For example, iodine provides significant advantages in clear visualization of lesions and enhanced CT imaging. However, some patients have allergic reactions to iodine contrast agents, and in mild cases, symptoms such as skin rash and itching may occur, while in severe cases, severe reactions such as anaphylaxis may occur. Traditional small molecule iodine contrast agents, such as iohexol and iopanoic acid, have small molecular weights and are easily and rapidly excreted from the body via the kidneys after injection, so they may cause some damage to the kidneys, especially for patients with renal insufficiency, the risk is greater.^64^ Gadolinium contrast agents have a certain degree of nephrotoxicity, especially linear gadolinium contrast agents, which may lead to serious kidney diseases,^65^ such as nephrogenic systemic fibrosis. Despite the low incidence of allergic reactions to gadolinium contrast agents, there is a risk of anaphylactoid reactions. Barium sulfate, which is widely used for gastrointestinal tract imaging, has a relatively high safety profile but still suffers from poor biocompatibility and is not suitable for specific populations.^66^ Consequently, clinicians must decide on the use of these agents based on the patient’s specific circumstances. Additionally, these contrast agents typically lack targeting functions.

#### 3.1.2 The Application of Nanomaterials in Imaging

**Table 2:** Summary Table of Nanomaterials

Common Nanomaterials	Advantages	Disadvantages	ref
SPIONs	1. Superparamagnetic	1. T2 negative contrast agent	(69−72)
	2. Excellent biocompatibility	2. Limited resolution	
	3. Multimodal imaging		
Au NPs	1. High X-ray absorption properties	1. Poor stability in vivo	(73, 74)
	2. Easy surface modification		
	3. Excellent biocompatibility and low toxicity		
Bi_2_S_3_ based nanomaterials	1. Strong near-infrared absorption and high efficiency of photothermal conversion, can be used for photothermal therapy	1. Potential security risks	(75, 76)
	2. Excellent CT imaging		
WO3	1. Excellent CT imaging	1. Poor biocompatibility	(77)
	2. Excellent photothermal performance		
LNPs	1. Excellent biocompatibility and degradability	1. Possible decrease in environmental stability in the body	(78−82)
	2. High drug loading capacity		
	3. Easily targeted modifications		
Dendritic macromolecules	1. High drug loading capacity	1. Complex metabolism in the body	(83)
	2. Excellent biocompatibility and water solubility		
	3. Easily targeted modifications		
Polymer micelles	1. Can integrate multiple imaging agents to achieve multimodality imaging and improve diagnostic accuracy	1. Vulnerability to in vivo distribution	(84)
	2. Excellent biocompatibility and stability		
	3. High drug loading capacity		
PBNPs	1. Multimodal imaging capability with MRI and photoacoustic imaging	1. Lack of targeting of unmodified nanoparticles	(85)
	2. Potential for photothermal imaging		
	3. Excellent biocompatibility and low toxicity		
	4. Easily surface modified and functionalized		
Biomimetic membrane nanoparticles	1. Excellent biocompatibility and stability	1. Complicated preparation	(86−92)
	2. Strong targeting properties		
	3. High drug-carrying capacity		

While conventional organic contrast agents may exhibit varying degrees of toxicity, some modified nanoparticles are often nontoxic, and at the same time can be endowed with a number of desirable imaging properties, making them ideal candidates for contrast agents.

Nanoparticles can be labeled with fluorescent dyes or radioactive isotopes for use in imaging techniques such as CT, MRI, and PET.

A significant role of nanomaterials in imaging is their function as probes for precise lesion area visualization. Targeted probes are commonly employed for this purpose.^67^ These probes typically consist of signal components and affinity components. The signaling component, such as radioactive nuclides, fluorescein, or paramagnetic atoms, generates imaging signals detectable by imaging devices. The affinity components, also known as targeted molecules, specifically bind to the target, such as antibodies or ligands.^68^

Nanomaterials widely used in AS can be mainly classified into metallic nanomaterials, organic nanomaterials and biological nanomaterials. Common metallic nanomaterials include superparamagnetic iron oxide nanoparticles (SPIONs), gold nanoparticles (Au NPs), cerium oxide (CeO_2_) nanoparticles, bismuth sulfide (Bi_2_S_3_) based nanomaterials, nano tungsten oxide (WO3) and so on. Organic nanomaterials include liposome nanoparticles (LNPs), dendritic macromolecules, polymer micelles, Prussian blue nanoparticles (PBNPs), etc. Biomimetic membrane nanoparticles are mainly some nanoparticles modified in a way that mimics cell membranes.

While general contrast agents can only monitor the overall morphology, size and other characteristics of the plaque, nanomaterials can observe the extent of the lesion at the molecular level. For example, surface-targeted modification of magnetic iron oxide nanoparticles can obtain information at the molecular level within the plaque, which is beneficial for MRI detection of AS.^93,94^ Dendrimers are a class of macromolecular compounds with a regular structure and monodisperse nature, with a typical symmetric core, inner shell and outer shell. The outer shell has multiple terminal functional groups that provide a wide range of space and binding sites for targeting molecules and therapeutic agents.^95,96^ More importantly, dendritic macromolecules are able to overcome the short half-life, poor specificity and high nephrotoxicity of traditional conventional imaging agents.^97,98^ Thus, it can be used as an excellent carrier for molecular imaging and contrast agents.

Nanomaterials such as these can be used in disease imaging to more accurately monitor disease states and can be effective in minimizing many adverse effects.

##### 3.1.2.1 Ultrasound

Ultrasound is a technique that uses high-frequency ultrasound waves to penetrate the human body and visualize its internal structures,^99^ with the significant advantage of no damage from ionizing radiation, and is therefore widely used in clinical practice as a low-cost, real-time, noninvasive and safe imaging tool. Although ultrasound imaging can reveal the basic morphological features of diseases, there are limitations in its resolution, especially when displaying tiny lesions or fine structures, which may be difficult to achieve the desired clarity.

To overcome this challenge, ultrasound molecular imaging has been developed to improve the sensitivity and specificity of ultrasound imaging. This technology greatly enhances the ultrasound signal in the lesion area by introducing targeted contrast agents, providing powerful support for early diagnosis of disease and real-time monitoring of treatment efficacy. These ultrasound molecular contrast agents typically consist of highly efficient carriers such as microbubbles and nanoparticles, whose surfaces are carefully modified to carry high-affinity molecules such as antibodies, peptides or small molecule drugs, ensuring that they can precisely target-bind to pathologically altered tissues or cells to provide accurate imaging of disease.

Triple-negative breast cancer (TNBC) is challenging to treat with immunotherapy alone due to the tumor microenvironment. It has been demonstrated that lactic acid produced by tumor cells affects tumor-associated macrophages (TAMs), promoting the conversion from M1 to M2 macrophages.^100−103^ Additionally, the unfavorable tumor microenvironment can impair the immune response, facilitating immune escape by tumor cells.^104^ Cisplatin can upregulate inflammation-related genes and increase the proportion of dendritic or macrophage cells, contributing to the formation of a more favorable microenvironment for tumor therapy. However, cisplatin also upregulates certain autophagy behaviors in tumor cells. Tumor autophagy is a key mediator of cytotoxic T-lymphocyte immune escape and can inhibit the production of immune cytokines. Therefore, inhibiting tumor cell autophagy may enhance the efficacy of immunotherapy. Chloroquine (CQ), a drug that inhibits autophagy by blocking the fusion of autophagosomes and lysosomes, can be used in combination with cisplatin to attenuate the increase in autophagy induced by cisplatin.

To enhance targeting and reduce adverse reactions, researchers utilized nanomaterials to load the drug and incorporated the imaging agent perfluoroethane (PFH)^105^ to assist with imaging. This approach facilitated phase change localization and imaging through ultrasound treatment.^106^ The drug was released smoothly when the nanoparticles were exposed to sonication and the hydrazone bonds were broken in the acidic environment. The HPLC results indicated that the release rates of Pt and CQ from the nanoparticles were significantly higher in the combination of sonication than in the absence of sonication, and showed a significant delayed release effect.

**Figure 4:**
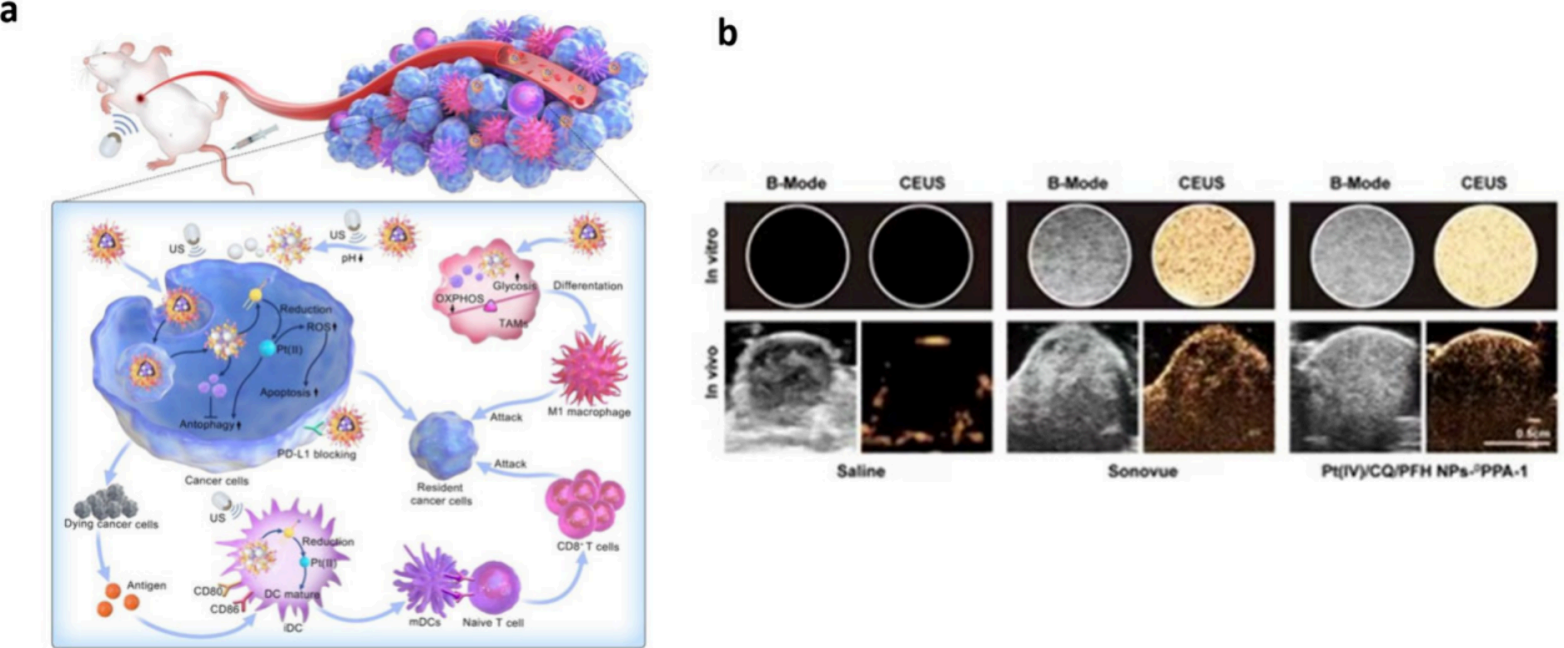
(a) The mechanism of Pt(IV)/CQ/PFH NPs-DPPA-1. (b) In vitro and in vivo imaging maps, showing the negative control, positive control, and experimental group from left to right. Reproduced with permission from reference (106). Copyright 2022 American Chemical Society.

PFH undergoes a phase transition in the presence of ultrasound, resulting in enhanced ultrasound visualization of nanoparticles. In the imaging effect evaluation experiment, the results indicated that the negative control group produced almost no signal, whereas both the experimental group and the positive control group exhibited strong echogenic signals and clear ultrasound images. These findings demonstrated that Pt(IV)/CQ/PFH NPs-DPPA-1 provides effective ultrasound imaging (Figure 4).

As shown in Figure 4b, the experimental group exhibited excellent imaging results.

In addition to imaging with ultrasound alone, researchers have also tried to combine ultrasound with other imaging modalities. Based on the potential of ultrasound as a source of energy to stimulate molecules to emit light, combined with the principle of acoustic luminescence, researchers have developed an innovative ‘ultrasonic luminescence imaging’ technology. The new imaging method uses ultrasound to stimulate fluorescent molecules to generate luminescent signals in vivo, enabling a new method of imaging with high-intensity optical signal output.^107^ Compared to conventional fluorescence imaging, this technique demonstrates multiple advantages with lower background noise, higher signal-to-noise ratio, better imaging sensitivity and deeper imaging depth. By implementing a two-step internal energy conversion process, the research team successfully screened trianthracene derivative-based nanoparticles (TD NPs), which achieved significant enhancement in in vivo ultrasound-induced luminescence imaging. The research data revealed that their ultrasound-induced fluorescence intensity not only surpassed that of acoustic luminescence, but also performed better in terms of signal-to-noise ratio, imaging sensitivity and imaging depth. The ultrasound-excited luminescent molecules developed in this study achieved a multifold enhancement in luminescence intensity compared to conventional aqueous acoustic luminescence signals. Compared to fluorescence imaging, the signal-to-noise ratio of ultrasound-excited luminescence imaging is significantly improved due to the absence of interference between the ultrasound signal and the optical signal emission, while maintaining high resolution and tissue penetration.

##### 3.1.2.2 MR

MRI contrast is usually generated by inducing water proton relaxation, and Gd is an ideal candidate for proton relaxation and therefore widely used in MRI.^108,109^ However, free Gd^3+^ is highly nephrotoxic and must be administered in its stabilized form to prevent serious human toxicity. Gd chelators can fulfill these conditions.^110^ The Gd chelator derivative Gd-DTPA can shorten the proton T1 relaxation time and improve blood brightness, and thus is widely used in MR diagnosis of vascular diseases.^111,112^ Based on the great damage caused by free Gd^3+^ to the human body, stable chelating agents can be chosen for the preparation of Gd contrast agents, which can firmly encapsulate Gd^3+^ and prevent it from freeing, thus reducing the toxicity. Chelators with high thermodynamic and kinetic stability can reduce the chance of Gd^3+^ dissociation from the chelator. In addition, the chelator structure can be optimized, such as increasing the number of ligands or introducing more stable ligands, thus improving the binding ability of the chelator to Gd^3+^. However, due to the small hydrodynamic size of small molecule Gd chelates and the short blood circulation time, there are still great challenges in high-resolution vascular imaging.^113^ Attachment of Gd chelates to the surface of nanoparticles increases relativity, which may enhance MRI contrast. Therefore, if gadolinium chelates are combined with functionalized nanomaterials, the imaging benefits of gadolinium can be effectively exploited and adverse effects can be reduced. For patients unable to use Gd chelates, functionalized nanomaterials modifying Gd chelators can present a safer alternative. Additionally, current research on nanoparticles often integrates targeting, imaging, and drug delivery functions, which are crucial for guiding clinical disease imaging and treatment.

**Figure 5:**
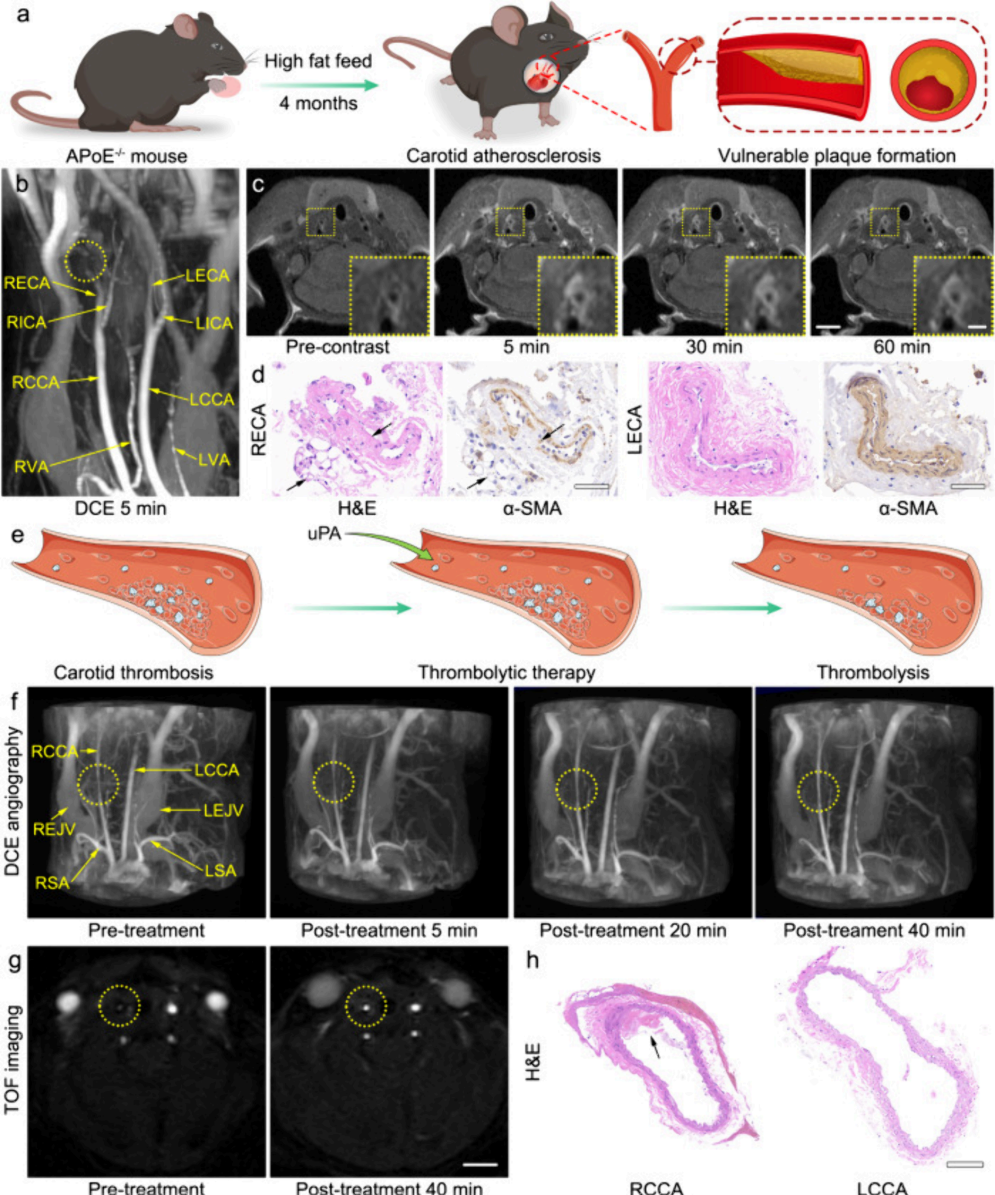
(a) Schematic drawing of the atherosclerosis plaque model establishment. (b) Representative PAA-Gd-enhanced 3D MR angiography at 5 min postcontrast, for showing the vascular stenosis site (yellow dotted circle). (c) Representative *T*_1_-weighted images of plaque section acquired pre- and at different time points postinjection of PAA-Gd. Triplicates were performed independently with similar results. (d) H&E and α-SMA staining of tissue slices from external carotid arteries. Triplicates were performed independently with similar results. (e) Schematic drawing of the thrombosis and thrombolytic therapy. (f) PAA-Gd-enhanced 3D angiography of mouse carotid thrombosis pretreatment (obtained at 5 min after PAA-Gd injection), and the real-time monitoring of thrombolytic therapy post-treatment of urokinase (uPA). (The carotid thrombosis was identified by yellow dotted circle). (g) Representative 2D time-of-flight (TOF) angiography of thrombus section acquired pre- and 40 min post-treatment of uPA. Triplicates were performed independently with similar results. (h) H&E staining of bilateral common carotid arteries. Triplicates were performed independently with similar results. The embedded scale bar of frame (c), (c) inset, (d), (g), and (h), corresponded to 2 mm, 0.5 mm, 50 μm, 2 mm, and 100 μm. Reproduced with permission from reference (114). Copyright 2023 PubMed Central.

For example, the researchers coupled DTPA and PAA to prepare a magnetic polymer carrier. Gd^3+^ was then cleaved with DTPA, resulting in a metal-chelating polymer called PAA-Gd.^114^ The ideal hydrodynamic size and structure gave it an appropriate blood half-life and low immunogenicity. After a series of experiments, it was determined that PAA-Gd exhibited a strong safety profile. And in a disease model of atherosclerosis in mice, the carotid artery, carotid vein, aortic arch and even vertebral artery were clearly visualized after injection of PAA-Gd in the tail vein. And fibrous stenosis could be seen in the middle of the common carotid artery (yellow dashed part), which indicated that the 3D vascular MRI contrast based on PAA-Gd could achieve precise targeting of the thrombus portion (Figure 5). To verify the MRI results, the experimenters performed histological studies on mice. Endothelial damage and thrombosis of aortic vessels could be detected by H&E staining maps, which coincided with the results of MRI, indicating that PAA-Gd can effectively improve the imaging ability of MRI.

Phosphatidylinositol (PS) residues externalized in apoptotic cells can trigger phagocytosis via the macrophage scavenger receptor pathway. Imaging plaques with PS-containing liposomes to mimic apoptosis is a promising approach. Researchers study PS-rich (Gd-PS) paramagnetic liposomes in atherosclerotic plaques.^115^ Similarly, a research team prepared a Gd-containing liposome containing CD36 ligand based on CD36 on the surface of macrophages, and showed through experimental results that this functionalized liposome not only highly accumulates in the area of plaque lesions, but also significantly improves the imaging clarity.^116^

**Figure 6:**
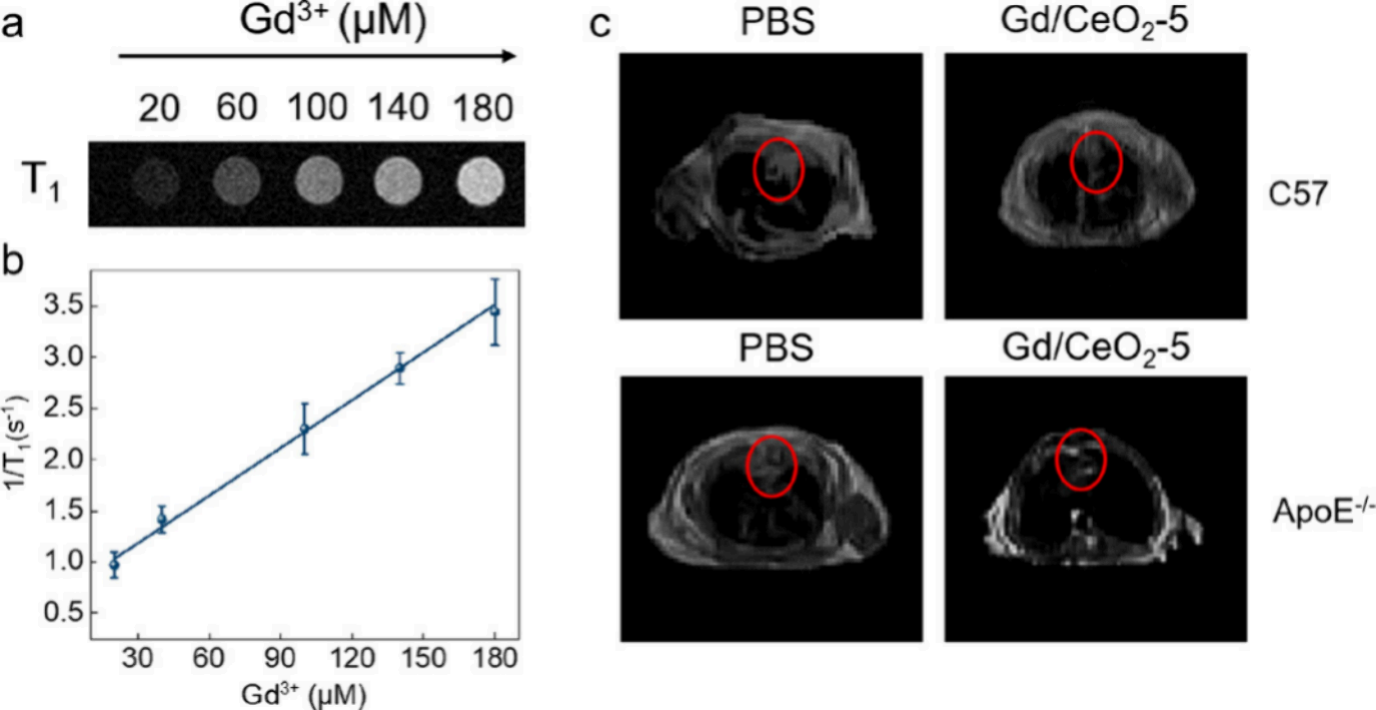
Imaging effect after Gd addition. (a) T1 images obtained after coincubation of cells with the nanoparticle at different Gd concentrations. (b) Linear plot of 1/T1 versus Gd concentration. (c) MR imaging of the nanoparticles applied to C57 and ApoE^–/–^ mice, respectively. Reproduced with permission from reference (122). Copyright 2023 American Chemical Society.

Ce is a key component of human glutathione peroxidase (GSH-PX) and plays a critical role in antioxidant activity. Doping Gd into CeO_2_ nanoparticles effectively increases the surface ratio of Ce^3+^, thereby simulating the activities of catalase (CAT) and superoxide dismutase (SOD). This enhancement allows for the effective clearance of reactive oxygen species (ROS),^117−121^ significantly alleviating vascular disease by reducing lipid accumulation in macrophages and lowering inflammatory factors, which helps inhibit the progression of atherosclerosis.^122^ Furthermore, Gd/CeO_2_ can act as a T1-weighted MRI contrast agent, providing sufficient contrast to distinguish plaque locations during live imaging (Figure 6). Due to the damaged vascular endothelium in the plaque region, it may have promoted a high degree of aggregation of nanoparticles in the plaque region, further enhancing the imaging treatment effect.

As shown in the figure, T1 imaging efficiency for this nanoparticle is positively correlated with Gd concentration, enhancing MR imaging effects in both in vitro and in vivo experiments.

Some nanomaterials not only serve as carriers but also possess physicochemical properties that aid in imaging or target specific cells in the body. SPIONs exhibit magnetic targeting properties and provide effective imaging in MRI. Liu Y synthesized a degradable amphiphilic polymer, PIA-g-PEG-g-DDA, grafted with polyethylene glycol via free radical polymerization. 5-Hydroxytryptamine (5-HT) was grafted onto PIA-g-PEG-g-DDA through an esterification reaction to produce 5-HT-g-PIA-g-PEG-g-DDA. This carrier specifically targets MPO within plaques.^123^ The carrier, coated on the surface of SPIONs, facilitates precise imaging of the lesion area. Carbon fluorescence quantum dots are adsorbed via electrostatic interactions to produce dual-mode imaging nanoprobes with T2 imaging and optical imaging functions. This nanocarrier not only offers precise targeted imaging but also features low toxicity and easy degradation, making it advantageous as both a contrast agent and a carrier.

##### 3.1.2.3 CT

Bismuth-based nanomaterials are highly sensitive to X-rays and hold significant potential for CT imaging and other applications. Studies have shown that inorganic bismuth-based materials, with their high atomic numbers and X-ray attenuation coefficients, are ideal contrast agents for CT imaging. Consequently, bismuth-based nanomaterials have promising prospects in imaging and tumor treatment.

Initially, bismuth was investigated as a protective agent to reduce radiation during CT examinations. Subsequent studies found that bismuth agents could reduce radiation in superficial areas without compromising image quality. Some bismuth-based nanoparticles, due to their high X-ray absorption and extended in vivo circulation time, can serve as alternatives to conventional iodine-based CT contrast agents. Bi_2_S_3_ nanoparticles prepared by Rabin’s team demonstrated minimal cytotoxicity compared to the same dose of free Bi^3+^, thereby ensuring high-quality CT imaging while reducing the risk of adverse effects associated with conventional contrast agents.^124^

**Figure 7:**
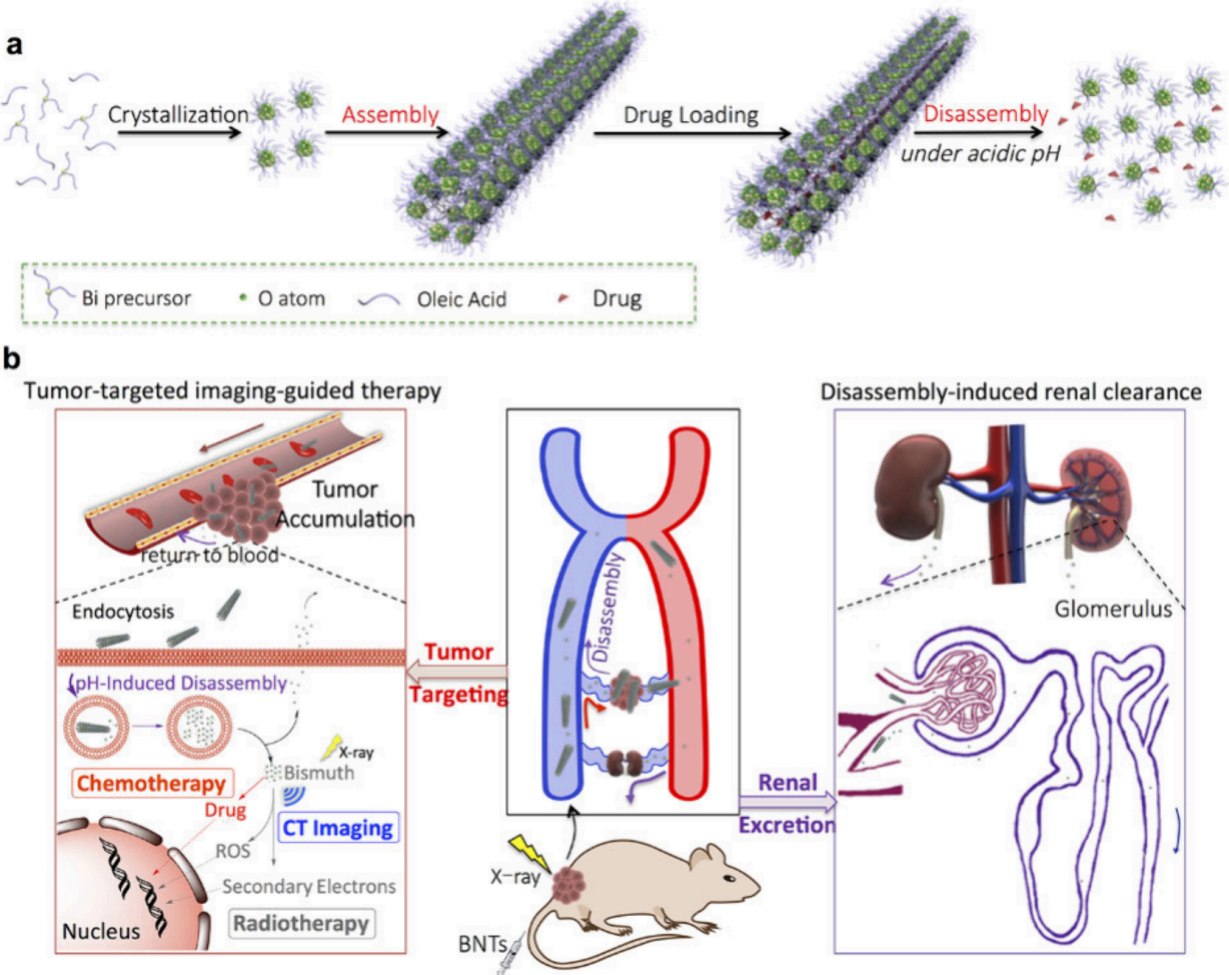
Schematic diagram of the synthesis and principle of action of BNTs. (a) Schematic illustration of the formation of BNTs. (b) Schematic illustration of tumor-homing of elongated BNTs, CT imaging-guided radio-/chemotherapy mediated by the BNTs/drug, and subsequent BNTs disassembly and renal clearance. Reproduced with permission from reference (124). Copyright 2018 American Chemical Society.

To significantly enhance the CT imaging effect of bismuth-based nanomaterials,^125^ researchers prepared ultrasmall (BiO)_2_CO_3_ nanoclusters (BNCs) from bismuth subcarbonate ((BiO)_2_CO_3_) nanotubes (BNTs) (Figure 7). The preparation considered two critical factors: X-ray attenuation and cyclic half-life. Experimental results demonstrated that BNTs exhibited strong targeting capability and an excellent plasma half-life.^126^ BNTs can be decomposed into BNCs in acidic environments, thus providing powerful imaging capabilities.

In vivo experiments showed that BNCs had a short plasma half-life and were rapidly cleared from the body, whereas BNTs remained in the target area for a longer duration and demonstrated superior imaging effects. CT imaging confirmed that BNTs provided better imaging capabilities compared to BNCs.

##### 3.1.2.4 Multimodal Imaging

With the growing recognition of the advantages of multimodal imaging, researchers have been developing nanoparticles that integrate multiple imaging modalities to further enhance imaging effects for disease treatment.^127,128^

**Figure 8:**
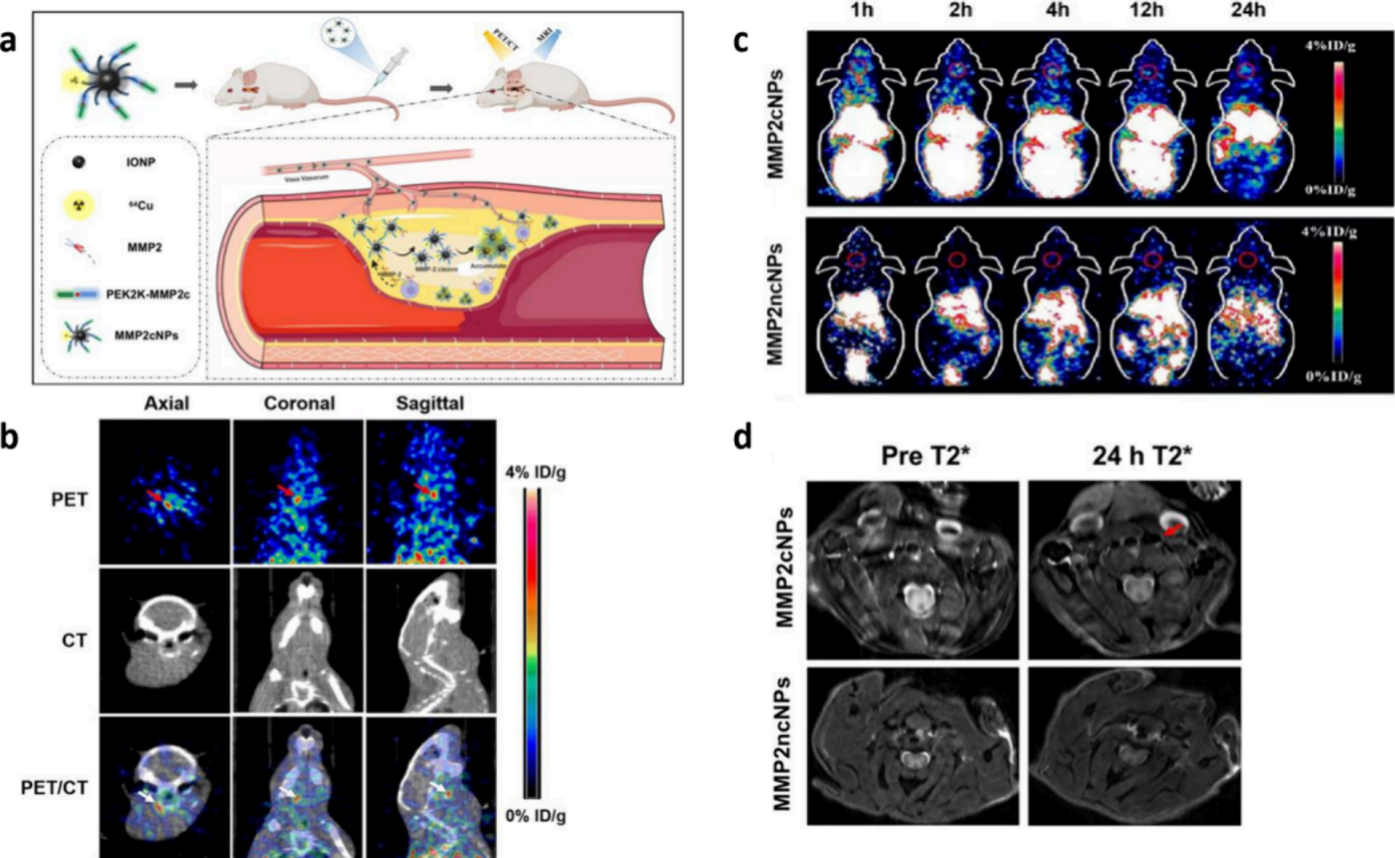
(a) The mechanism of MMP2cNPs. (b) The representative in vivo PET, CT, and PET/CT images of mice models with carotid atherosclerotic plaques at 4 h after injection of MMP2cNPs. (c) Coronal PET images of mice models with carotid atherosclerotic plaques at 1 h, 2 h, 4 h, 12 and 24 h after injection of MMP2cNPs or MMP2ncNPs. (d) In vivo CL57/BL6 mice with carotid atherosclerotic plaque MRIs obtained before and 24 h after administration of MMP2cNPs or MMP2ncNPs at a dose of 10 mg iron/kg (red arrow, cross section of the carotid artery). *International Journal of Nanomedicine***2022**, *17*, 6773–6789. Originally published by and used with permission from Dove Medical Press Ltd. Reproduced with permission from reference (130). Copyright 2022 PubMed Central.

Tu’s team developed a contrast agent capable of cleaving nanoparticles with multifunctional imaging effects based on matrix metalloproteinases in macrophages (Figure 8a). The researchers derivatized iron oxide nanoparticles with 1,4,7-triazacyclononane-1,4,7-triacetic acid (NOTA), labeled them with ^64^Cu as a nuclear tracer, and modified the MMP-2 cleavage peptide with PEG2000.^129^ This led to the creation of a multimodal imaging nanoparticle named ^64^Cu-NOTA-IONP@MMP2cPEG2K (MMP2cNPs).^130^ These nanoparticles demonstrated multimodal precision imaging capabilities, as confirmed by combined experimental results.

Atherosclerotic lesions in the left carotid artery were clearly visible 4 h after injection of MMP2cNPs, showing high contrast with the low background in the contralateral artery (Figure 8b). In contrast, no PET signal was detected in the carotid plaques of mice injected with MMP2ncNPs, indicating that cleavable nanoparticles offer significant in vivo imaging capabilities and prolonged action.

Imaging was clearer following the injection of MMP2cNPs. MMP2cNPs, being less water-soluble than MMP2ncNPs, exhibited slower diffusion in the plaques, resulting in extended in vivo retention and enhanced imaging selectivity and sensitivity (Figure 8c, 8d).

Additionally, researchers can optimize the lifespan, tissue distribution, biocompatibility, and targeting of nanoparticles by modifying their surface chemistry, size, and shape, thereby improving their utilization.^131,132^

For instance, researchers evaluated lipid, macrophage, and cholesterol crystal properties in atherosclerosis-prone plaques using media depolarization index based on catheter-based polarized bright–dark optical coherence tomography (PS-OCT).^133^ This study demonstrated that EMDI has a high plaque recognition rate and that PS-OCT provides excellent imaging results.

### 3.2 Application of Nanomaterials in the Treatment of Atherosclerosis

#### 3.2.1 Traditional Treatment Methods

The treatment of AS can generally be categorized into medications and other therapeutic approaches (Table 3).

**Table 3:** Advantages and Disadvantages of Traditional Treatment Methods

Treatment methods		Advantages	Disadvantages	ref
Drugs	Lipid-Lowering Drugs	1. Therapeutic effectiveness	1. Side effects	(134)
		2. Multiple targets	2. Drug interactions	
	Anti-Inflammatory Drugs	1. Significant anti-inflammatory effect	1. Liver function impairment	
		2. Improvement of endothelial function	2. Renal injury	
		3. Antithrombotic and antioxidant effects	3. Rhabdomyolysis	
		4. Enhanced therapeutic effect		
	Vasodilators	1. Improvement of blood flow	1. Potential for tolerance	
		2. Relief of symptoms	2. Drug interactions	
		3. Prevention of complications	3. Side effects	
Others	CABG surgery	1. Improved blood supply	1. Invasive procedure	(135, 136)
		2. Long-term survival	2. Potential complications	
		3. Wider applicability	3. Limited graft availability	
			4. Reoperation risk	
	PCI	1. minimally invasive	1. Restenosis	
		2. Effective treatment	2. Need for long-term medication	
		3. Wide applicability	3. Limited durability	
	RA	1. Effective removal of calcified plaque	1. Potential complications	
		2. Treatment of complex lesions	2. Restenosis	
		3. Distinction between plaque and healthy tissue	3. Need for adjunctive therapies	
		4. Advanced technology		

Additionally, methods such as coronary radiation therapy are also available. The choice of specific treatment should be tailored to the patient’s individual condition to achieve the best therapeutic outcomes.

#### 3.2.2 Application of Nanomaterials in Treatment

**Figure 9:**
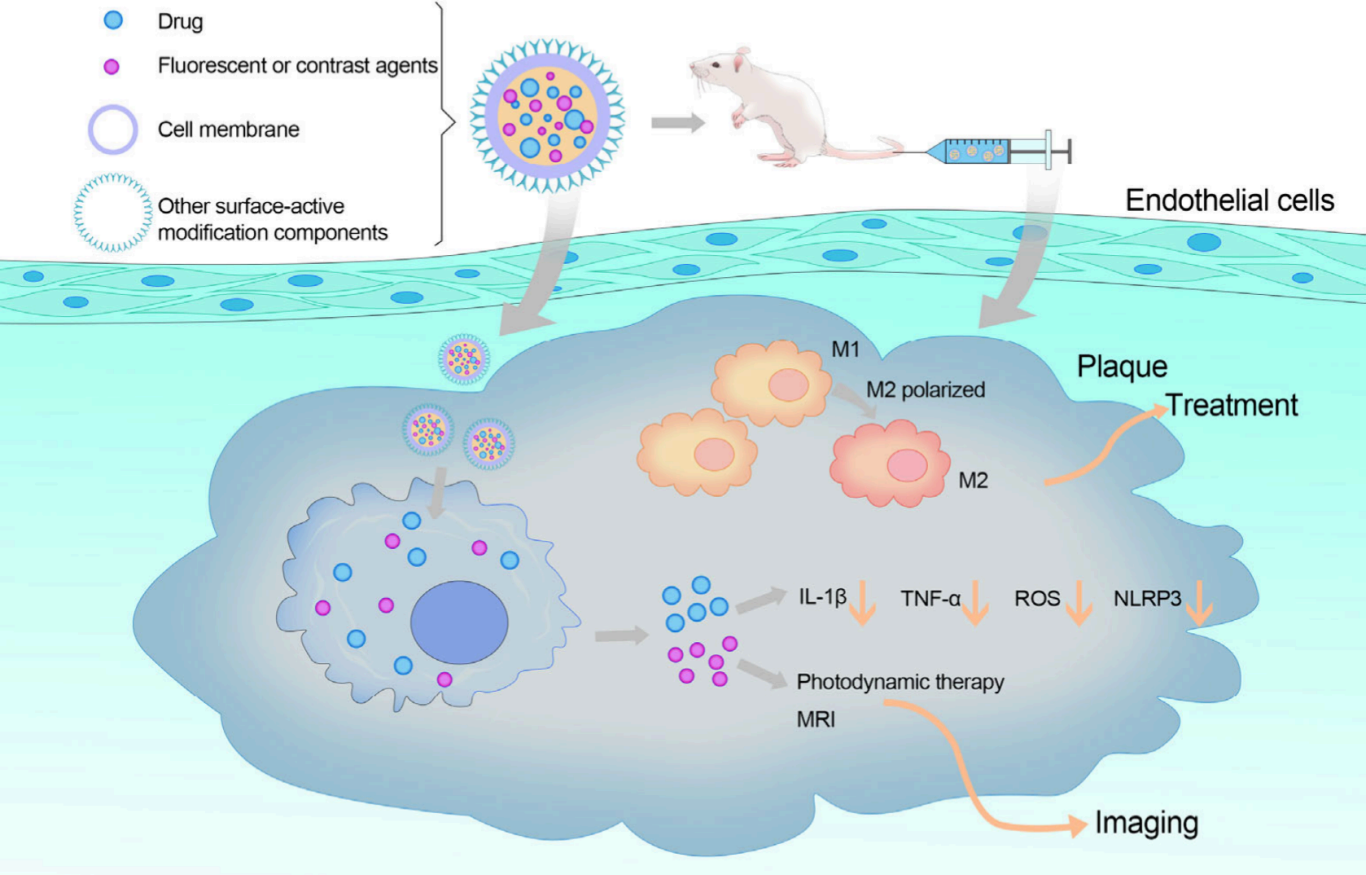
Mechanisms of nanomaterials. Common and desirable nanomaterials generally function as carriers while also providing targeting and imaging capabilities for precision therapy. Besides drug delivery, nanoparticles often encapsulate fluorescent or contrast agents. To avoid phagocytosis and prolong their half-life, nanoparticles may incorporate components with high biocompatibility, such as macrophage membranes. Additionally, bioactive substances on the surface of nanoparticles can enhance precision therapy through high affinity to the target. In animal experiments, nanoparticles reach the plaque site following tail vein injection and release various components that influence inflammatory factors in the plaque environment. Some nanoparticles can even promote macrophage polarization to the M2 phenotype, contributing to disease treatment. For imaging, the inclusion of contrast agents enhances MRI efficiency, while fluorescent agents significantly improve fluorescence imaging, resulting in clearer visualization of diseases.

Nanomaterials are incorporated into nanomedicines using nanotechnology, entering the body through various forms of nano dispersion systems to exert their therapeutic effects (Figure 9). With advancements in nanotechnology, nanoparticles, nanoliposomes, solid lipid nanoparticles (SLNs), nanospheres, nanocapsules, nanoemulsions, and polymer micelles are among the popular nanomaterials.^137^

Nanomaterials as drug carriers can enhance the absorption of poorly soluble oral drugs, deliver drugs with poor bioavailability or efficacy via conventional routes more accurately to the target, and reduce waste and adverse reactions. Additionally, nanomaterials can serve as contrast agents to target lesion locations, thereby facilitating clinical treatment.^138^ Key characteristics of nanocarriers generally include: Affinity: This includes the affinity between the nanocarrier and the loaded drug, as well as the affinity with the in vivo target. Stability: This refers to the binding stability of drugs and targets, as well as the stability of the internal environment. Effective nanocarriers maintain stable efficacy over time, exhibit slow metabolism and easy excretion, and do not cause biological accumulation.

Nanomaterials not only function as probes for targeted imaging but also integrate imaging, drug delivery, and targeted localization. They exhibit varying effects at different stages and reactions in the treatment of AS.

##### 3.2.2.1 Inhibiting Inflammatory Response

During the development of AS, the release of inflammatory factors promotes the transformation of monocytes into macrophages, which then become foam cells.^139^ This inflammatory reaction contributes to vascular endothelial damage, lipid metabolism disorders, and plaque expansion in AS. Given the numerous inflammatory targets, inhibiting inflammation is a critical aspect of AS treatment.

In AS plaques, CD4+ helper T (Th) cells are the primary adaptive effector cells, responding to presented apolipoproteins. Th1 cells secrete factors that stimulate macrophage transformation and AS formation, while Th2 cells promote the production of IL-4, IL-5, IL-13, and IL-33. IL-4 promotes AS, whereas IL-5, IL-13, and IL-33 have anti-inflammatory effects and inhibit AS formation. Macrophages activated by different stimuli primarily differentiate into M1 and M2 phenotypes. M1 macrophages, driven by Th1 responses, produce inflammatory factors, while M2 macrophages, influenced by Th2 responses, exhibit anti-inflammatory effects. Therefore, polarizing M1 macrophages to M2 macrophages can effectively counteract inflammation and support AS development.

The sulfonylurea derivative glyburide, a medication for type 2 diabetes, exhibits anti-inflammatory properties. It can polarize proinflammatory M1 macrophages into M2 macrophages and inhibit T cell immune responses. However, glyburide’s hydrophobic nature and instability in the circulatory system pose challenges. To address this, a research team developed PLGA nanoparticles loaded with anti-inflammatory liposomes and coated with red blood cell membranes.^140^ These nanoparticles were characterized both physically and chemically, and their drug release profiles were studied after confirming biocompatibility. RT-PCR results demonstrated a reduction in inflammatory gene expression, including NLRP3, IL-1β, IL-18, caspase-1, caspase-8, and caspase-9, indicating potential for cellular uptake, inhibition of inflammatory genes, and prevention of plaque formation in rabbit AS models.

During AS onset, a significant number of inflammatory cells are recruited near white blood cells (WBCs). To exploit this, white blood cell membranes were used to encapsulate magnetic nanoclusters (MNCs) with excellent superparamagnetism. These MNCs were embedded with the anti-inflammatory drug simvastatin and modified with the targeted apolipoprotein A-I mimetic peptide L-4F (AP) to produce MNC@M-ST/AP. This nanoparticle targets early AS lesions, achieves precise drug release, blocks further inflammatory responses, and delays or prevents plaque formation and diffusion.^141^ Additionally, the membrane shell allows for the loading of hydrophobic drugs, addressing issues of poor water solubility. The embedded AP inhibits the assimilation of ox-LDL, facilitates TC outflow, and demonstrates excellent safety in pathological and clinical biochemical analyses.

Due to its excellent targeting and immune escape capabilities, the macrophage membrane has recently emerged as a crucial biological membrane material for nanoparticles. Studies have demonstrated that macrophage membranes contain CD47 protein and integrin α4/β1 receptors. The CD47 protein effectively prevents cells from being engulfed, while integrin α4/β1 binds to macrophages via endothelial adhesion factors on vascular endothelial cells, thereby facilitating plaque targeting. Building on this concept, our team designed and prepared macrophage membrane-coated colchicine nanoparticles, modified with CD47 and integrin, named MMM/COL NPs.^142^ The CD47 protein protects the nanoparticles from being engulfed, while integrin guides them to precisely target macrophages infiltrating plaques. Colchicine is then released to exert its anti-inflammatory therapeutic effects. In vitro and in vivo experimental results demonstrate that these nanoparticles effectively reduce foam cell formation, downregulate pro-inflammatory factor expression, exhibit excellent plaque targeting, stabilize plaques, and show good safety for major organs.

##### 3.2.2.2 Inhibiting Oxidative Stress

Oxidative Stress (OS) is a state in which there is an imbalance between oxidative and antioxidant effects in the body, tending to oxidize, resulting in an inflammatory infiltration of neutrophils, an increase in the secretion of proteolytic enzymes, and the production of large amounts of oxidative intermediates. Oxidative stress is a negative effect produced in the body by free radicals and has been implicated as an important factor in the exacerbation of AS.

ROS are byproducts of oxidative metabolism in the body with strong oxidizing properties. ROS can oxidize LDL to form ox-LDL, which damages endothelial cells, induces monocyte adhesion and chemotaxis to the endothelium, and promotes macrophage foam cell formation, thereby contributing to plaque development. Consequently, utilizing nanotechnology to efficiently eliminate free radicals is a major therapeutic approach for AS.

In recent years, iron oxide-cerium oxide core–shell nanoparticles have emerged as promising materials for addressing ROS-related inflammatory diseases such as AS.^143^ Research indicates that these nanoparticles can effectively clear ROS and provide excellent MRI performance in vitro.

To counteract ROS effects, Liu R developed ROS-responsive and scavenging material TPCD, which was loaded with the anti-inflammatory drug Darapladib that selectively inhibits lipoprotein-associated phospholipase A2 (LP-PLA2), creating TPCD-DA NP through nanotechnology.^144^ In vitro cell experiments revealed that the nanoparticles effectively reduce intracellular ROS levels and oxidative stress-induced cellular aging. In vivo studies demonstrated that TPCD-DA NP significantly reduced the area of aortic plaques and effectively modulated atherosclerosis development. Additionally, the nanoparticles improved the reduction in gut microbiota abundance caused by a high-fat diet, thus reducing aortic plaque area.

Based on the mechanism by which epigallocatechin gallate (EGCG) blocks fatty acid-induced expression of caveolin-1 and cyclooxygenase-2 (COX-2), EGCG can act as an antioxidant, while Distearoylphosphatidylcholine (DSPC) serves as the main carrier to load Simvastatin (SIM), forming liposome nanoparticles (SE LNPs).^145^

Experimental results have demonstrated that these nanoparticles exhibit ROS responsiveness, antioxidant properties, antiapoptotic effects, and promote M2 macrophage polarization.^146^ Additionally, the liposomal formulation provides sustained drug release, thereby prolonging the drug’s in vivo retention time and enhancing its therapeutic efficacy.

NF-E2 related factor 2 (Nrf2) is a transcription factor activated by high levels of ROS, which regulates downstream target genes encoding detoxification factors, antiapoptotic proteins, antioxidant enzymes, and drug efflux transporters. Activation of Nrf2 leads to the reduction of oxidative damage and repair of damaged proteins.^147^ To selectively deliver Nrf2 activators, CDDO-Me (a potent Nrf2 activator) is encapsulated into nanoparticles to produce antioxidant-activated nanoparticles (ARAPas).^148^ In vitro experiments have shown that ARAPas significantly induce the activation of the antioxidant enzyme HO-1 in macrophages. This effectively provides anti-inflammatory effects, inhibits disease progression due to inflammation, and plays a crucial role in managing atherosclerosis.

Based on the inflammatory mechanism of AS pathology, Liu Shun designed a multifunctional drug delivery system targeting macrophages, with sensitivity to pH and ROS. One component is a nano micelle targeting macrophages with ROS sensitivity (HASC@Cur).^149^ Another component is a multifunctional nanocarrier responsive to hyaluronic acid and pH, designed for chemical and photothermal synergistic therapy for AS (Cus@DMSN-N = C-HA). This system has successfully achieved precise drug delivery and controlled release, as well as synergistic chemical and photothermal therapy. Compared to single-targeted treatment methods, this multifunctional drug delivery system has demonstrated significant advancements in both innovation and therapeutic efficacy.

Our team synthesized a nanoparticle combining targeted delivery and ROS responsiveness. This nanoparticle uses a biodegradable polymer micelle composed of polyethylene glycol-poly(tyrosine-ethyl oxaloacetate) (PEG-Pyr-EO) encapsulated in hyaluronic acid (HA), carrying Simvastatin (SIM). This formulation aims to inhibit macrophages and reduce ROS levels to treat atherosclerosis.

**Figure 10:**
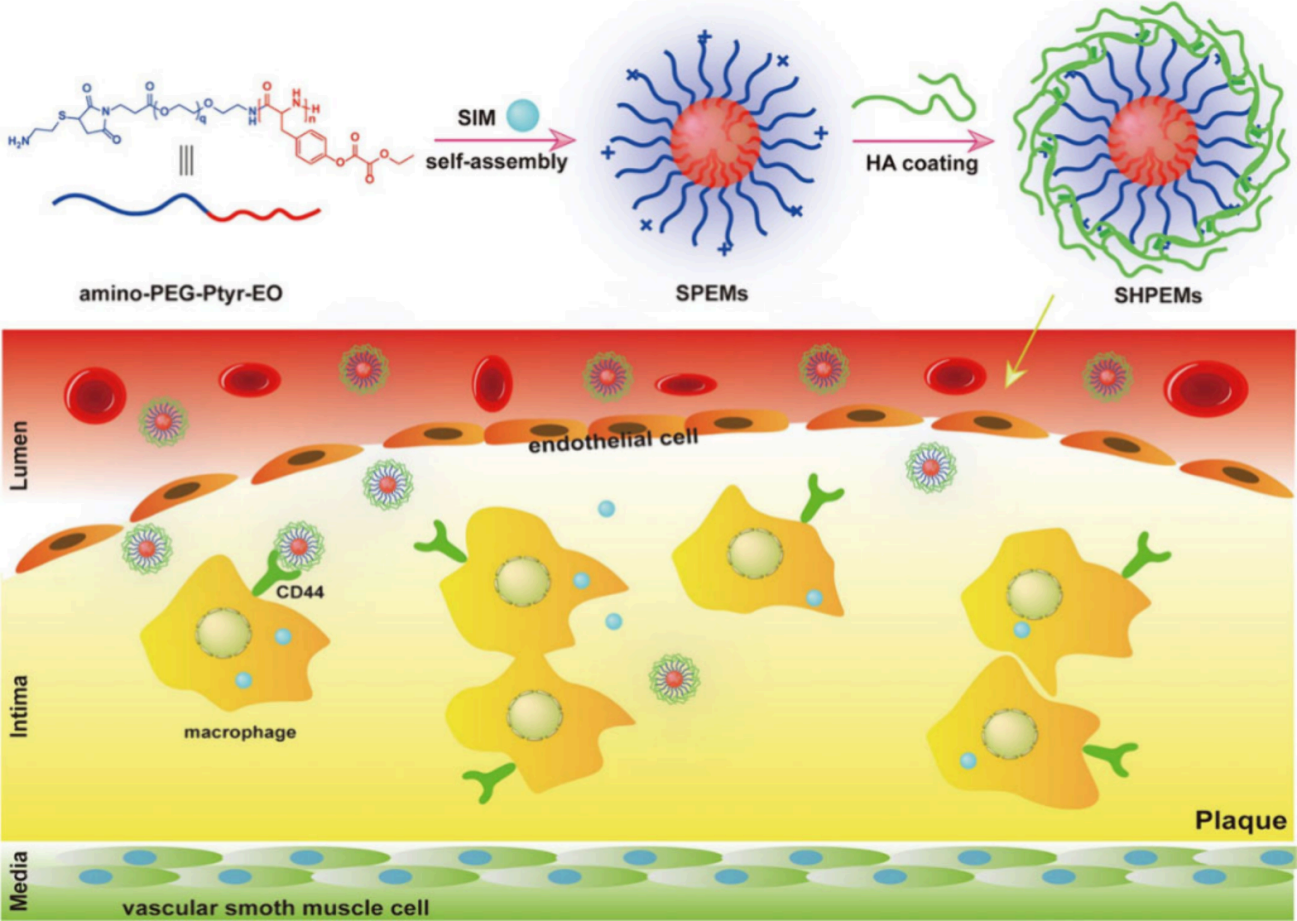
Preparation route and targeted delivery process of SIM from SHPEMs. SIM: simvastatin; HA: hyaluronic acid; SHPEMs: SIM-loaded amino-PEG-Ptyr-EO micelles; SHPEMs: SIM-loaded HA-coated amino-PEG-Ptyr-EO micelles. Reproduced with permission from ref (150). Copyright 2020 PubMed Central.

HA targets inflammatory macrophages due to CD44, a specific marker that is highly expressed in inflammatory regions. As a result, HA enhances targeting to inflammatory sites in plaques. PEG-Pyr-EO reacts with H_2_O_2_, consuming it in a concentration-dependent manner, which means that the drug delivery rate increases with higher concentrations of H_2_O_2_. This specificity minimizes the impact on normal tissues, thereby improving safety (Figure 10).

**Figure 11:**
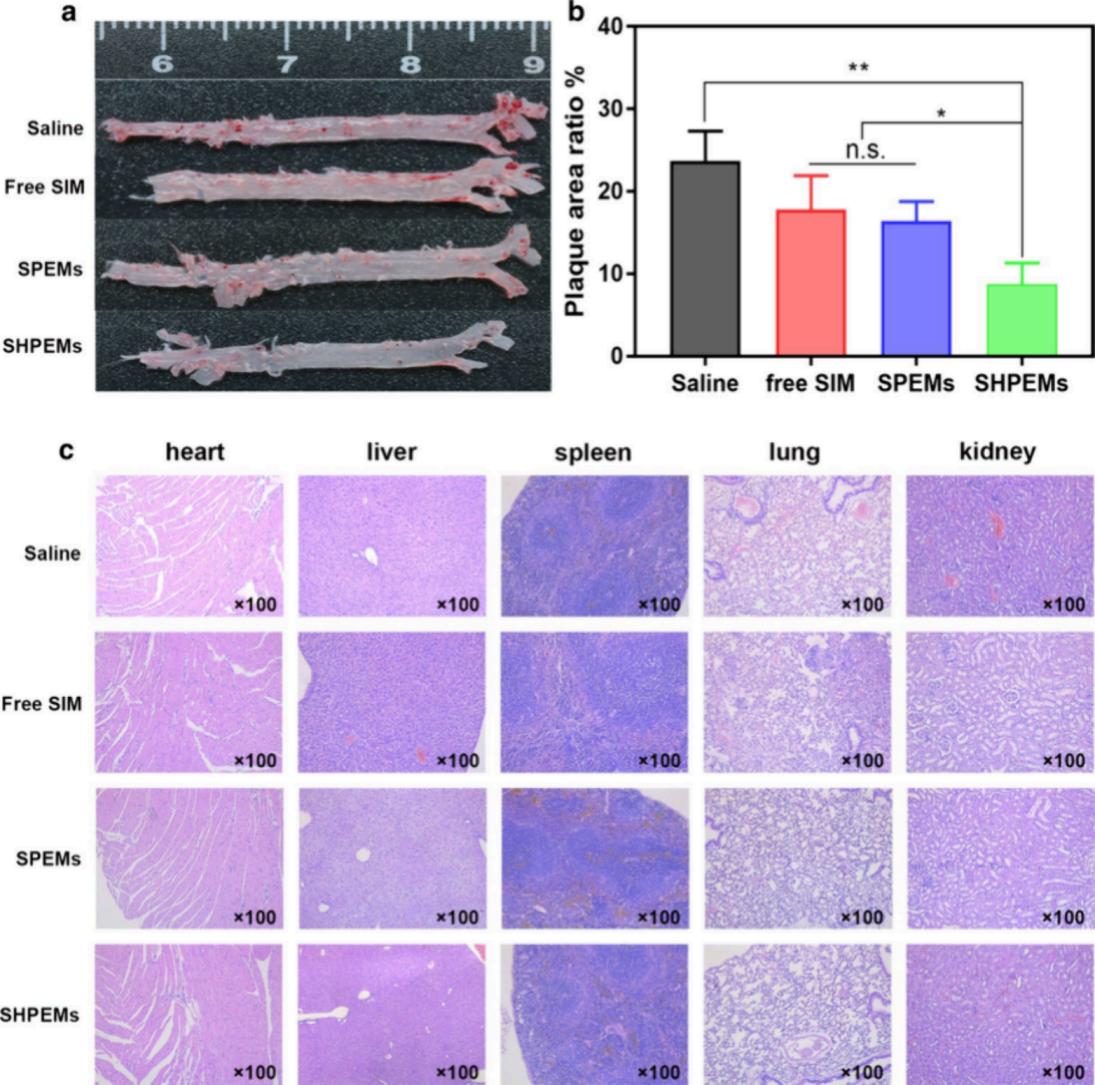
Results of pathological experiments in mice. (a) Ratio of plaque area to total area. (b) H&E staining of the heart, liver, spleen, lung, and kidney from mice in each group. (c) SIM: simvastatin; SPEMs: SIM-loaded amino-PEG-Ptyr-EO micelles; SHPEMs: SIM-loaded HA-coated amino-PEG-Ptyr-EO micelles. Reproduced with permission from ref (150). Copyright 2020 PubMed Central.

Simultaneously, the SIM released at the lesion site can maximize antioxidant effects, enhance synergistic treatment, and improve efficacy. Our results show that the nanoparticles effectively reduced cholesterol content in atherosclerotic mice and produced significant therapeutic effects without causing adverse reactions (Figure 11).^150^

In the ApoE^–/–^ AS mouse model, dandelion polysaccharides were administered as treatment. After treatment, levels of HDL-C, SOD, and GSH-PX in the serum increased, while LDL-C, malondialdehyde (MDA) were significantly reduced.^151^ This indicates that dandelion polysaccharides have antioxidant effects in AS and hold potential as an important drug for its treatment. This advancement could significantly enhance the field of antioxidant drugs, potentially elevating AS treatment methods to a higher level.

Wei Tao’s team synthesized BPNSs-PEG-S2P/R based on black phosphorus nanosheets (BPNSs) as the main body, modified S2P peptide, and loaded with Resolvin D1 (RvD1), which promotes the process of cytosolic burial.^152^ The study demonstrated that the BPNSs possessed an excellent ROS scavenging effect,^153,154^ and the S2P had a high affinity with stablin-2 expressed on the macrophages’ surface in the plaques.^155^ The experimental results showed that BPNSs-PEG-S2P/R significantly reduced ROS levels and elevated the expression levels of antioxidant factors such as glutathione (GSH) and SOD. It also facilitated oxidative phosphorylation and fatty acid oxidation at the plaques, thus promoting cytosolic burial, reducing necrotic cores, and stabilizing plaques.

##### 3.2.2.3 Adjusting Ferroptosis

Ferroptosis, an iron-dependent form of nonapoptotic cell death, has an important role in the development of cardiovascular disease.^156,157^ The key to ferroptosis is the iron-catalyzed peroxidation of phospholipids (PLs) containing polyunsaturated fatty acids (PUFA–PLs), leading to the lethal accumulation of lipid peroxides in the cell membrane and cell membrane rupture, which ultimately leads to cell death.^158−160^ Ferroptosis plays a significant role in exacerbating AS. Therefore, exploring the mechanism of ferroptosis is a crucial step in treating AS.

The mechanisms of ferroptosis can be categorized into two main processes: the Fenton reaction induced by free iron and the lipid peroxidation process catalyzed by fatty acid enzymes.^161^ During transport, Fe^3+^ in the body is reduced to Fe^2+^ by the STEAP3 protein. Fe^2+^ then reacts with peroxides to form peroxide bonds, which oxidize lipids, ultimately leading to plaque formation.

The clearance of intracellular peroxides primarily depends on the action of glutathione peroxidase 4 (GPX4).^162^ GPX4 uses GSH as a substrate to reduce lipid peroxides to normal phospholipid molecules. Consequently, GPX4 is a key target for many therapeutic agents aimed at inhibiting ferroptosis.

Ferroptosis is closely related to lysosomal function: activating iron autophagy, such as molecular chaperone-mediated autophagy, promotes the degradation of GPX4, leading to increased lipid peroxidation and ferroptosis.^163^ Additionally, the release of lysosomal cathepsin (CTSB) can also trigger ferroptosis.^164^ Furthermore, the accumulation of lysosomal iron or nitric oxide strongly promotes ferroptosis.^165^

During oxidative phosphorylation, increased ROS in mitochondria further contributes to ferroptosis, which can be inhibited by mitochondria-targeted antioxidants or enzymes.^166^ Thus, iron death inhibitors can be categorized into those targeting iron ions, ROS, lysyl oxidase (LOX), etc.^167^

Regulating ferroptosis is crucial in treating AS. Researchers have employed nanomaterials to study ferroptosis in vivo, aiming to control the pathological progression of AS. Nanoparticles such as MoS2 can cause lysosomal dysfunction, leading to Fe^2+^ release, lipid peroxidation through the Fenton reaction, and subsequent ferroptosis. Zero-valent iron (ZVI) induces the oxidation of ZVI to Fe^2+^, assisting in oxidative reactions, promoting mitochondrial lipid peroxidation, and regulating ferroptosis. Iron-based nanoparticles (FeNPs) can release Fe^2+^ or Fe^3+^ in acidic lysosomes, inducing cellular ferroptosis.^168^ Nanoparticles similar to Au increase the expression of the intercellular adhesion protein E-cadherin, thereby inhibiting ferroptosis.^169^

Based on the principle of the Fenton reaction, we prepared nanoparticles called HFTNPs through chemical treatment. HFTNPs is mainly composed of three parts: dopamine-modified hyaluronic acid(HD), ferricion, Tannic acid (TA). As mentioned, HA has a high affinity for CD44 on macrophage membranes, making it a targeted approach.^170,171^ TA serves both as an iron carrier and a reductant of iron ions. Ferricion is a key component in the Fenton reaction and also functions as an important contrast agent for MRI.^172^

Our results indicate that the nanoparticles are highly concentrated in inflammatory macrophages, enhancing ROS production and promoting the apoptosis of inflammatory macrophages without impairing normal cell activity.

**Figure 12:**
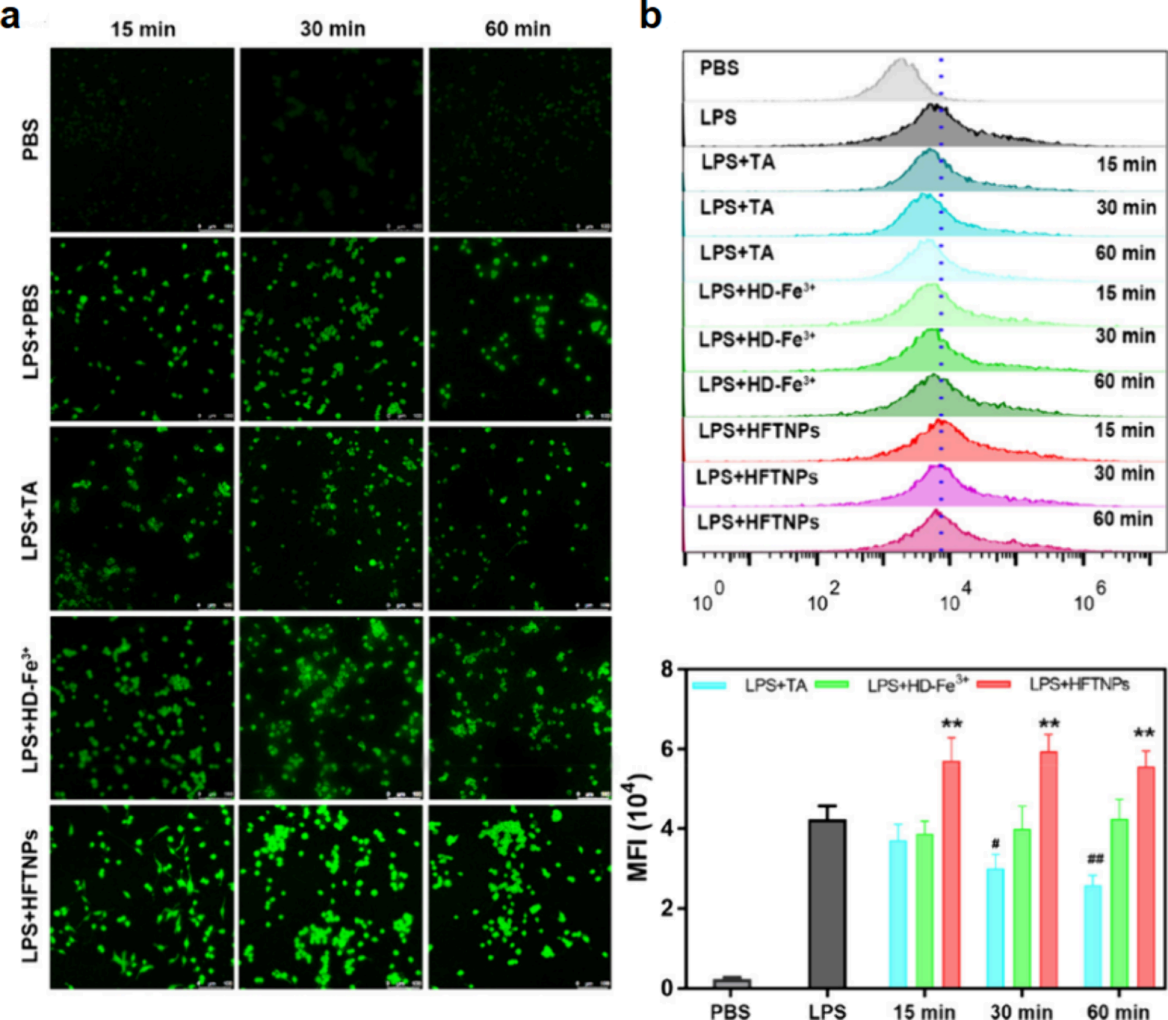
Rate of intracellular ROS production with different treatments over time. (a) Representative fluorescent images of LPS-activated Raw264.7 macrophages incubated with TA, HD-Fe^3+^, and HFTNPs for 15, 30, and 60 min, respectively. Raw264.7 macrophages were treated with PBS as control. TA: 29.4 μM, iron: 117.6 μM. Scale bars: 100 nm. (b) Quantitative analysis of LPS-activated Raw264.7 cells incubated with PBS, TA, HD-Fe^3+^, and HFTNPs for 15, 30, and 60 min by flow cytometry. LPS + HFTNPs vs LPS: **p < 0.01; LPS + TA vs LPS: ^*#*^*p <*0.05 and ^##^*p* < 0.01. Reproduced with permission from ref (172). Copyright 2021 American Chemical Society.

ROS levels increased after LPS treatment, while ROS production decreased in the presence of TA (Figure 12). However, with HFTNPs, ROS levels increased, inducing inflammatory macrophage death via the Fe response and exerting an antiatherosclerotic effect. The regulation of the ferroptosis process and related cellular molecules can also serve as a novel approach for treating AS.

##### 3.2.2.4 Endothelial Protection

It is well-known that the initiation of atherosclerosis, as well as the key pathophysiological process of vascular restenosis after interventional procedures, involves damage to the vascular endothelium. Therefore, accelerating the process of reendothelialization of damaged vessels and restoring the normal function of endothelial cells are essential to promote positive regression of such diseases. Endothelial progenitor cells (EPCs), derived from hematopoietic stem cells, combine the antigenic characteristics of both stem cells and endothelial cells, and as the precursor of endothelial cells, they play multiple roles. EPCs not only stimulate endothelial cell proliferation and differentiation through the release of cytokines, and at the same time inhibit the proliferation of smooth muscle cells, thus regulating the process of reconstruction of the vascular network; they are also able to differentiate into mature endothelial cells, which participate directly in the construction of vascular network. EPCs play a crucial role in maintaining endothelial integrity and repairing damaged vessels.^173,174^ When exposed to cardiovascular disease risk factors, EPCs biological function declines, with a subsequent decrease in the ability to repair vascular damage.^175^ Therefore, self-optimized EPCs or transplantation of functionally improved EPCs may be able to make up for this deficiency. Meanwhile, studies have shown that statin lipid-lowering drugs can improve the functions of EPCs differentiation, proliferation, adhesion and migration, and inhibit its apoptosis, thus repairing the damaged endothelium and improving the vascular endothelial function.^176^

Based on this concept, Liu proposed to transplant EPCs endocytosed with pitavastatin nanoparticles into rats with carotid endothelial injury to repair the injured blood vessels.^177^ Experimenters isolated spleen-derived EPCs from rats and treated them with pitavastatin nanoparticles and injected them into carotid artery-injured rats. Eventually, it was found that inhibition of pitavastatin nanoparticle-treated EPCs could promote reendothelialization of injured blood vessels and significantly inhibit intimal hyperplasia of injured blood vessels. These results suggest that this endothelial repair-based therapy is promising for the treatment of atherosclerosis or restenosis after PCI.

The integrated stress response (ISR) of endothelial cells, triggered by a complex stress environment in the cardiovascular system and inflammatory reactions, contributes to atherosclerosis. ROS produced by endothelial cells activate proinflammatory factors,^178^ stimulate monocytes to differentiate into macrophages, and lead to foam cell formation through lipid phagocytosis, thus inducing plaque formation. Changes in endothelial permeability and increased Ca^2+^ release result in the calcification of vascular smooth muscle cells (VSMCs), accelerating atherosclerosis progression. Endothelial damage can also cause rupture of the AS plaque, promoting thrombus formation. SOD, CAT, and GPX effectively clear ROS, alleviating or preventing ROS-induced peroxidation reactions and hindering the development of AS.

When endothelial cells are subjected to stress, there is often an increase in HSP expression. Research indicates that HSP27, through its interaction with the NF-κB pathway, can stabilize plaques and reduce inflammation levels. HSP70 is hypothesized to protect against the toxic effects of modified LDL particles on cells, making it a potential target for reducing oxidative stress. The presence of HSP60 antibodies in AS patients may exacerbate endothelial damage and inflammatory reactions in vascular walls, further advancing AS. Thus, HSP60 is a critical focus for anti-AS strategies and may serve as a basis for developing anti-AS vaccines. Evidence suggests that HSP60/HSP65 has the potential to prevent or at least reduce the severity of atherosclerosis.^179^

In LDL receptor knockout (LDLR^–/–^) mice, HSP65 tolerance induced by Mycobacterium significantly reduced lesions, particularly in lymphocytes with reduced responsiveness to HSP60 and increased IL-4 production. This suggests that IL-4 may play a protective role in atherosclerosis development. In mice nasally administered HSP65, there was a decrease in macrophage positivity in the aortic arch, an increase in IL-10 production, a reduction in CD4+ T cell count, and a decrease in IFN-γ production, alongside an increase in transforming growth factor-β (TGF-β) generation. Different HSP subtypes play a crucial role in the treatment of AS.^180^

In the early stages of AS, inflammatory reactions damage the endothelial cells of blood vessels, leading to increased expression of adhesion molecules such as vascular cell adhesion molecule-1 (VCAM-1) and intercellular adhesion molecule-1 (ICAM-1). These adhesion molecules facilitate the extravasation of lymphocytes and monocytes from the bloodstream to the subendothelial layer. However, studies have shown that brown adipose tissue (BAT)-derived neuregulin 4 (Nrg4) inhibits endothelial inflammation through the ErbB4-Akt-NF-κB pathway. Nrg4 reduces leukocyte homing and macrophage aggregation in plaques, attenuates adhesion reactions, and improves plaque stability, thereby effectively protecting the endothelium.^181^

**Figure 13:**
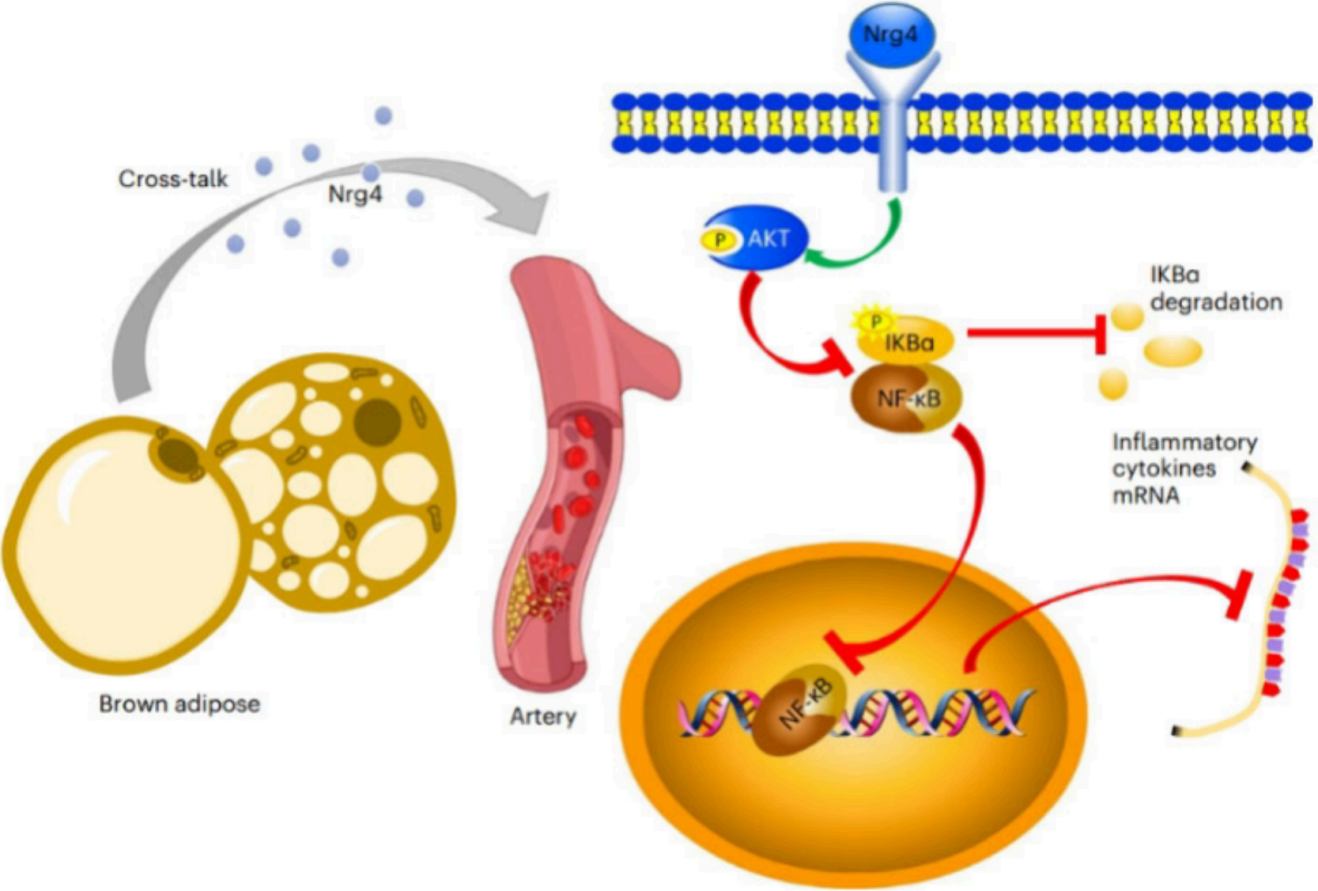
Schematic representation of Nrg4’s protective role in atherosclerosis via the ErbB4-Akt-NF-κB signaling pathway. Reproduced with permission from ref (181). Copyright 2022 PubMed Central.

Studies have also shown that Nrg4 reduces the number of macrophages and improves inflammation in Crohn’s disease, indicating its anti-inflammatory effects.^182^ This finding introduces new targets and treatment methods for anti-AS, expands the approaches and research perspectives in AS treatment, and encourages researchers to explore previously uncovered areas (Figure 13).

##### 3.2.2.5 Stem Cell Utilization

In addition to conventional cellular molecules, many researchers have attempted to apply nanoparticles in stem cell therapy.

One of the main problems with cardiac regeneration after myocardial infarction is myocardial cell death, which is replaced by noncontractile fibrous scar tissue instead of newly formed myocardial cells; Weakening the ability of organs to contract normally increases the risk of further heart attacks. Researchers have been trying to use regenerative medicine technology to grow new muscles in damaged hearts using pluripotent stem cells (PSCs). Currently, it is believed that human pluripotent stem cells (hPSCs) can serve as a possible pathway for regenerative medicine to treat AS. Due to the fact that these cells can now be stimulated to differentiate into various types of cells, it is believed that they can be directly injected into the site of injury, stimulating the reconstruction of damaged blood vessels. Research has found that stem cell transplantation improves the vascular function of recipient animals by stimulating the formation of neointimal SMCs. Therefore, this treatment method is expected to generate new myocardial cells.^183^ Recent research has mainly focused on effectively targeting specific sites of stem cells, one of which is to use echogenic liposome as stem cell carriers.^184^ In addition, it was found that procoagulant liposomes can prevent the pathogenesis of atherosclerosis.

The use of nanomaterials not only improves the retention and proliferation of implanted stem cells, but also enables imaging and tracking of stem cells in vivo, accelerating the discovery of the driving mechanisms of stem cells and their applications in medicine. For example, magnetic nanoparticles (MNPs) have been successfully used to separate and sort stem cells from various types of cell mixtures, with time-saving and economical characteristics; Quantum dots have been used for molecular imaging and tracking of stem cells. Carbon nanotubes (CNTs) and fluorescent carbon nanotubes have also been proven effective.

However, tracking stem cells in the body remains a major obstacle. Therefore, introducing ultra bright contrast agents is an optimization method.

Mesenchymal stem cells (MSCs) can differentiate into various cell types, including muscle, blood, blood vessels, fat, and bone cells. We have developed a plasma active nanoplatform for tracking MSCs after renal transplantation in mice.^185,186^ This platform relies on photoluminescence in principle and is composed of peptide functionalized gold nanoparticles (TAT-GNS). Through experiments, it has been found that the nanoplatform has better intracellular stability and sensitivity in vitro, and can cross the vascular barrier in vivo, with transferability. This platform can provide better tracking tools for stem cell therapy research and promote the development of diagnostic and therapeutic methods for cardiovascular diseases.

##### 3.2.2.6 Others

In the routine treatment of AS, the low effective utilization rate of drugs remains a pressing issue. Some drugs suffer from low permeability and absorption in the small intestine or are digested by enzymes in the internal environment, making it difficult for them to penetrate the biofilm and gastrointestinal mucus layer. Consequently, the bioavailability (F) of some oral drugs is very low, ranging from 0.1% to 14%, as seen with amikacin, gentamicin, neomycin, and carbendazim.

In the treatment of AS, taking the antiplatelet drug aspirin as an example, its oral bioavailability is about 40% −50%, which is much higher than the above drugs. However, after passing through the gastrointestinal first pass effect, the effective dosage of aspirin that truly acts in the body is greatly reduced, and the rate of action is slower. As a nonselective cyclooxygenase (COX) inhibitor, aspirin has no significant inhibitory effect on both COX-1 and COX-2, So it is inevitable to have an impact on the gastrointestinal tract, kidneys and other parts, inducing adverse reactions such as abdominal pain and gastrointestinal bleeding.^187^ Moderate aspirin can inhibit the synthesis of arachidonic acid (AA), the synthesis of thromboxane A2 (TXA_2_), and platelet activation by inhibiting COX-2. However, excessive intake of aspirin can lead to a decrease in prostaglandin (PGI_2_) production due to its stronger COX inhibitory effect on the vascular wall compared to platelet inhibition, PGI_2_ and TXA_2_ have physiological antagonism, which can lead to thrombosis. For AS patients, it can worsen vascular embolism and even cause myocardial infarction. Therefore, in such situations, the application of nanomaterials greatly reduces the occurrence of such adverse reactions.^188^

Nawzat reviewed the literature on the impact of Saudi Arabian nanocarriers on the oral bioavailability of drugs. In the study, Group A received drugs modified with various nanocarriers, including SLNs, polymer-based nanoparticles (such as polymer nanoparticles and polymer micelles/dendritic macromolecules), SNEEDS/nanoemulsion, liposomes/precursor liposomes, and nanostructured lipid carriers (NLCs). The bioavailability was represented by the area under the curve (AUC).^189^ The data showed that nanomodification of drugs and their loading into nanocarriers significantly improved oral bioavailability compared to unmodified drugs. This suggests that nanomaterials can enhance drug bioavailability through various nanoformulations. Due to their excellent biocompatibility, ability to carry both lipophilic and hydrophilic drugs, and targeted drug release capabilities, SLNs are among the most widely used nanocarriers.

Additionally, the application of nanomaterials to macromolecular drugs has recently become a research hotspot.

The Nano Drug Delivery System (NDDS) for proteins and peptides represents a significant advancement in the pharmaceutical field.^190^ By integrating molecular imaging with nanomaterials, this system offers vast development potential, particularly for delivering drugs through mucosal systems and utilizing antigen delivery methods as a novel vaccination strategy.

Macromolecular drugs are often prone to degradation in biological environments due to their complex structures, and their ability to cross biological barriers is limited.^191^ Research has demonstrated that nanomaterials can enhance the targeted delivery of oral macromolecular drugs using various nanocarriers, such as chitosan-encapsulated nano systems,^192^ liposomes,^193,194^ lipid nanocarriers primarily in the form of SLNs,^195−197^ biomimetic nano systems,^198^ and self-dispersed ionic liquid-based nanostructures,^199^ all of which show promising bioavailability. Additionally, chitosan nanoparticles can be administered nasally to achieve therapeutic effects.^200^

Conventional noninjection routes often face challenges in reaching target organs due to gastrointestinal mucosa, resulting in low drug bioavailability and adverse reactions. To address this, researchers have developed molecularly modified nanocarriers. These modified nanocarriers exhibit enhanced surface properties, such as targeting, gating, and pH sensitivity. They can specifically recognize receptors, increase retention time in the mucus layer or penetrate it rapidly, and open tight junctions in the gastrointestinal epithelial cell layer. This enables the precise and efficient delivery of drugs to target sites, significantly reducing drug loss.^201^ Moreover, some modified nanoparticles can enhance the permeability of gastrointestinal epithelial cells to carriers, thereby improving drug absorption in the small intestine and increasing oral bioavailability.

Compared to traditional synthetic drug treatments, natural drugs or nutritional supplements generally have fewer toxic side effects. Therefore, alternative artificial drugs can be explored as supplementary treatments to reduce adverse reactions and prolong patient survival.^202^

Research has shown that commonly used nanoparticles for AS imaging and treatment, including SPIONs, Au NPs, polymer nanoparticles, and LNPs, can effectively serve as carriers for nutrient delivery.^203^

## 4 Conclusions

By combining molecular imaging with nanomaterials, sensitive and targeted nanoprobes can be developed. Imaging technology can effectively detect the progression of AS before the onset of the disease, allowing for early intervention. During AS progression, nanomedicines loaded onto nanocarriers can target the lesion site, releasing drug particles at the specific location to achieve optimal efficacy, which has significant implications for clinical AS treatment.

Molecular imaging involves using imaging techniques to visualize specific molecules at the tissue, cellular, and subcellular levels, reflecting molecular-level changes in living conditions to qualitatively and quantitatively analyze their biological behavior.^204^ In essence, molecular imaging is a research discipline that applies molecular biology to traditional medical imaging techniques for tracking and treating living cells within organisms.

The integration of nanomaterials with modern molecular imaging technology has made a significant impact on disease imaging and treatment. Additionally, the use of nanomaterials in drug delivery has markedly improved the bioavailability of poorly water-soluble and orally ineffective drugs. Targeted administration with nanomaterials has also reduced adverse drug reactions and improved safety while maintaining effectiveness. Although substantial research exists on nanomaterials in the context of tumors both domestically and internationally, exploration in cardiovascular diseases remains underdeveloped. Therefore, this review aims to summarize the current research on the application of nanomaterials in imaging and treating AS plaques, both domestically and internationally, to advance the application and development of nanomaterials in the cardiovascular field. Although research on nanomaterials in cardiovascular diseases is growing, most techniques remain focused on preclinical studies, and several issues persist. For example, some nanocarriers may be recognized and cleared by the immune system,^205^ metabolic mechanisms may not be fully understood,^206^ and intravascular biomimetic robots can be affected by hemodynamics, complicating timely targeting. Consequently, research on nanomaterials for AS still faces significant challenges. The development of more convenient, efficient, and safe nanomaterial-based imaging and treatment technologies, and the effective translation of basic research into clinical practice, remains crucial.
